# Associations between child marriage and reproductive and maternal health outcomes among young married women in Liberia and Sierra Leone: A cross-sectional study

**DOI:** 10.1371/journal.pone.0300982

**Published:** 2024-05-20

**Authors:** Taylor Reisz, Kelly Murray, Anastasia J. Gage

**Affiliations:** Department of International Health and Sustainable Development, Tulane University School of Public Health and Tropical Medicine, New Orleans, Louisiana, United States of America; University of the Western Cape, SOUTH AFRICA

## Abstract

**Background:**

Child marriage has been associated with a range of negative maternal and reproductive health outcomes. This study explored these associations in Liberia and Sierra Leone and examined how child marriage intersected with other measures of social disadvantage.

**Methods:**

Data were derived from 631 and 1,325 married or cohabitating women aged 20–24 interviewed in the 2019–2020 Liberia and 2019 Sierra Leone Demographic and Health Surveys, respectively. Analyses were stratified by country. Regression models examined associations between age at first marriage (<15, 15–17, and 18+ years) and reproductive and maternal health outcomes, as well as interactions between child marriage and measures of social disadvantage. Multivariable regression results were presented as adjusted odds ratios and 95% confidence intervals.

**Results:**

Over half of currently married/cohabitating women aged 20–24 in Liberia (52%) and Sierra Leone (54%) married before age 18, and over one in 10 married before age 15. In both countries, after adjusting for other factors, being married before the age of 18 was significantly associated with early fertility, high fertility, and low fertility control. Associations were particularly strong among women who first married before age 15. In Liberia, women who married at age 15–17 had significantly lower odds of skilled attendance at delivery and institutional delivery if they lived in the North Central region. Sierra Leonean women who married before age 15 had lower odds of institutional delivery and lower odds of four or more ANC visits if they lived in the North Western region.

**Conclusion:**

This study found clear associations between child marriage and negative reproductive health outcomes in Liberia and Sierra Leone, with stronger associations among women married in early adolescence. Child marriage and region of residence intersected to shape young women’s access to skilled attendance at birth and institutional delivery. These findings call for further investigation and targeted intervention.

## Introduction

Child marriage, according to the United Nations (UN), is any union, forced or voluntary, where at least one partner is under age 18 [1]. Child marriage is widely considered a human rights violation. Across the globe, in the five years preceding 2022, child marriage had decreased by roughly 10%, but the economic and societal stress imposed by the COVID-19 pandemic, such as school closures and service disruptions, has put even more girls at risk [2]. In the next decade, up to 10 million more girls are projected to become child brides, on top of the 100 million who were already at risk of child marriage before the pandemic began [2]. While boys are impacted by the practice, the prevalence among girls is six times as high [3]. According to the latest available data, one in five women currently aged 20–24 were married or in union before age 18 [2], compared to one in 21 men in this age group, on average [4]. (For brevity, “married” in this analysis also encompassed women who were not married but living with their partner). In 2021, across sub-Saharan Africa, 35% of young women were married in adolescence [2]. In West and Central Africa, 37% of women aged 20–24 were married before age 18, and 12% were married before the age of 15 [5]. According to the latest country data from the Demographic and Health Surveys (DHS) (Liberia 2019–2020 and Sierra Leone 2019), about one in four Liberian [6] and nearly one in three Sierra Leonean women aged 20–24 were married before their 18^th^ birthday [7]. About 6% of young women in Liberia were married before age 15 [6], compared to about 9% in Sierra Leone [7].

Not only is child marriage associated with consequences such as poor educational opportunities [8, 9] and physical violence [10, 11], it also has clear maternal and reproductive health consequences, including inadequate antenatal care (ANC) utilization [12–14], unskilled delivery [13], adverse pregnancy outcomes [15], unintended/unplanned pregnancy [16, 17], rapid repeat childbirth, and pregnancy termination [14, 16]. Studies have shown that child marriage also negatively impacts young girls’ mental health, due in part to social isolation [18]. Furthermore, research has determined that childbearing during adolescence, a potential consequence of child marriage, can increase maternal morbidity as it carries a higher risk of complications such as eclampsia and infections [19, 20].

For Liberia and Sierra Leone, these associations were of particular concern, given already-high maternal mortality rates: From 2000 to 2020, World Bank data showed there was a 1 in 35 lifetime risk of maternal death in Liberia, and 1 in 52 in Sierra Leone [21]. The same data showed maternal mortality ratios in Liberia and Sierra Leone were 652 and 443 deaths per 100,000 live births, respectively [21]. The lowest rates in the world number were in the single digits, for example, three deaths per 100,000 live births in Australia and two deaths per 100,000 live births in Norway [21]. Given these associations and the persistence of child marriage in West Africa, this study sought to analyze maternal and reproductive health outcomes in the two West African nations to inform targeted health interventions that could mitigate the health impacts of child marriage.

Geographic disparities in maternal healthcare were also notable. For example, skilled attendance at delivery among women in Liberia was much lower in rural areas (79%) compared with urban areas (89%) [22]. The percentage of births delivered in a health facility ranged from 72% in the South Central region 91% in the North Central region [22]. Also, rural women in Sierra Leone were more likely than urban women to have the recommended four or more ANC visits (83% vs 73%) [7]. The percentage of institutional deliveries ranged from 92% in the Eastern province to 67% in the North West [7].

Our research contributes to the current academic literature in two ways. One is by determining whether there were differences in the association between child marriage and reproductive and maternal health outcomes in Liberia and Sierra Leone. Secondly, our study examined how child marriage intersected with certain measures of social disadvantage, such as poverty and region, to affect maternal and reproductive health outcomes.

## Materials and methods

### Selection criteria

**Fig 1:**
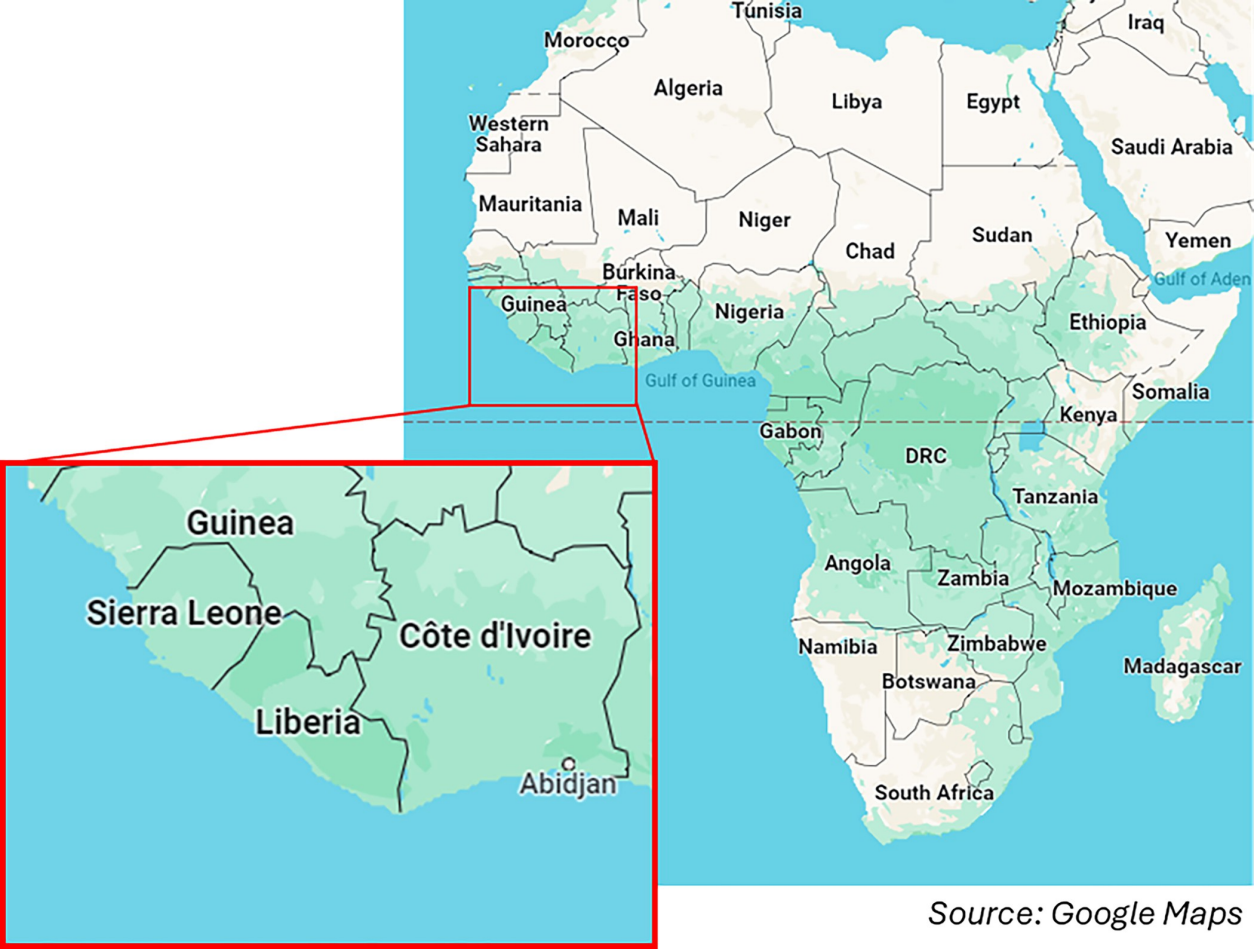
Map showing geographical location of Liberia and Siera Leone.

Liberia and Sierra Leone (see Fig 1), which share a border, also share a history of being established as colonies for freed slaves. In 1787, the British established a naval base in Freetown, the present-day capital of Sierra Leone, to intercept slave ships and act as a home for freed slaves [23]. In 1822, the U.S. founded Liberia to send formerly enslaved people from the Americas [24]. The two countries also rank near the bottom of 191 countries on the United Nations Human Development Index, with Liberia at 178 and Sierra Leone at 181 [25]. Liberia and Sierra Leone also share some ethnic groups, including the Mende and the Mandingo [26, 27].

More importantly, the two nations also share a history of stressors, including protracted civil conflicts and disease outbreaks. The Liberian Civil War spanned from 1989–2003, and the Sierra Leone Civil War lasted from 1991–2002. A 2023 study looked at the relationship between maternal and child health service coverage and armed conflict in sub-Saharan Africa, and found that conflict negatively impacted health service coverage [28]. It also found high-intensity conflicts increased the negative impact on early ANC and institutional delivery. A 2015 qualitative study found that poor access to maternal health services and poor maternal health outcomes were thought to be because of attacks on health facilities, medical supply looting, targeted killings or abductions of health providers, and other factors [29]. Additionally, a 2023 study used data from the DHS of West Africa and compared it with the locations of armed conflict. The researchers found that when conflict actors involved tactics like sexual violence and girl recruitment, the probability of becoming a child bride rose by 12% to 18% [30]. In addition, the 2014 Ebola outbreak, which was concentrated in Guinea, Liberia, and Sierra Leone, strained families financially and forced school closures. During that time, aid officials and survey respondents reported more families and girls turning to child marriage to alleviate their economic hardship [31–33].

Additionally, as in dozens of other countries around the world, Liberia and Sierra Leone have conflicting laws that do not fully protect children from the harmful practice [34]. In Liberia, the legal age of marriage is 18 for females and 21 for males, but a 16-to-17-year-old female or a male between the ages of 16 and 20 can marry with parental or guardian consent [35]. Sierra Leone’s Child Right Act of 2007 states, “The minimum age of marriage of whatever kind shall be eighteen years” [36]. However, the country’s Customary Marriage Act of 2009 allows child marriage with parental or guardian consent [35]. Other mechanisms that contribute to the continuation of child marriage include poverty, gender inequality, religious and cultural norms, and lack of education [37]. It is important to examine and compare these associations in places with histories of conflict, economic shocks, and conflicting laws around child marriage.

**Fig 2:**
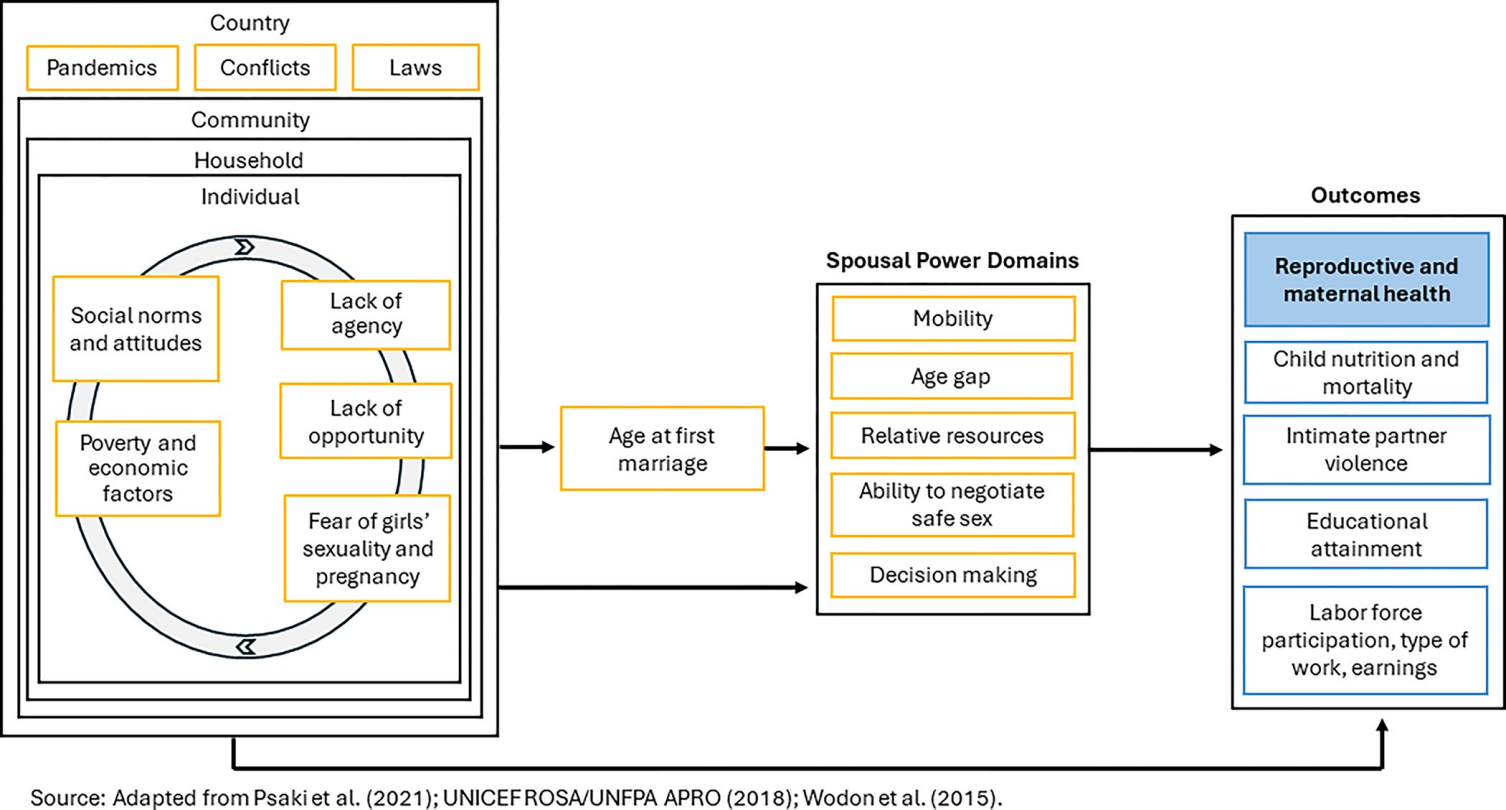
Conceptual model for the drivers and consequences of child marriage. Adapted from Psaki et al., 2021 [38] and the United Nations Children’s Fund and United Nations Population Fund (2018) [39].

Fig 2 highlights five main hypothesized social and economic drivers of age at first marriage (see lefthand side of conceptual model) that are situated within an individual’s household and community context, according to Psaki et al., (2021) [38]. Psaki et al. (2021) argue social norms and attitudes and poverty and economic factors “precede” the other three main drivers because they are “part of the larger social context in which decisions about marriage are made” [38]. These five main drivers, hypothesized by the United Nations Children’s Fund and United Nations Population Fund (2018), encompass “micro- and macro-level factors” including household size, religion, location, gender, birth order, access to information and communication technology, social policies, demographics, and women’s standing in society [39]. The conceptual model also denotes the larger country context in which these drivers are situated, with pandemics, wars and conflicts, and conflicting laws as drivers of age at first marriage, and the interconnectedness of factors at different levels of the social environment. The spousal power domains (second box from the right) are directly impacted by age at first marriage. According to existing research, child marriage is associated with reduced mobility (getting permission to seek medical help for self), a larger spousal age gap [14], a larger gap in relative resources between spouses (e.g., education), a reduced ability for the woman to negotiate safer sex [40], and reduced shared decision making [41].

The outcomes listed on the righthand side of the conceptual model include both individual and generational consequences of child marriage. These outcomes are also hypothesized to be directly influenced by the country, community, household, and individual-level drivers. The solid blue box labeled Reproductive and maternal health represents the outcomes of interest of the current study. The arrows in the model represent directionality of the hypothesized impact of the various drivers, with five main measures of social disadvantage at the country, community, household, and individual level influencing both age at first marriage as well as spousal power domains and reproductive/maternal health outcomes directly.

### Data sources

This study used secondary cross-sectional data from the most recent DHS completed in Liberia (2019–2020) and Sierra Leone (2019). The surveys were nationally representative and collected data on respondents’ characteristics, including fertility and fertility preferences, family planning, maternal and child health, and other topics. Our analyses were limited to 631 Liberian and 1,325 Sierra Leonean women aged 20–24 who were currently married or cohabiting at the time of the survey and had no missing data. For the analyses of maternal health outcomes, the data were further restricted to those whose most recent birth occurred in the five years preceding the survey.

### Variable definitions

#### Outcomes

We examined the following outcomes/dependent variables among women aged 20–24. All the dependent variables were binary (yes vs. no). Building on definitions used by Godha et al. [16] in their analysis of child marriage in four South Asian countries, we analyzed five reproductive health outcomes:

**Early fertility** measured whether the respondent reported giving birth within the first 12 months of marriage.

**High fertility** measured whether the respondent reported having had three or more live births, based on DHS standards for women married in adolescence.

**Low fertility control** indicated that the respondent reported having at least one live birth within 24 months of a previous birth, to enable comparison with prior studies. This indicator, also called “rapid repeat pregnancies,” increases negative health and socioeconomic consequences for young women and adolescents [42]. We do note the WHO’s recommended minimum birth-to-birth interval is 33 months [43].

**Unwanted/mistimed pregnancy** measured whether the respondent reported having an unwanted or mistimed pregnancy in the past five years (and included a baby who was wanted later or not at all).

**Modern contraceptive use** measured whether the respondent reported currently using at least one form of modern contraceptives, as outlined by the DHS: female or male sterilization, contraceptive pill, intrauterine contraceptive device (IUD), injectables, implants, female condom, male condom, emergency contraception, lactational amenorrhea method, standard days method, and other country-specific modern methods or methods mentioned by the respondent (but not menstrual regulation or abortions) [44].

We analyzed three maternal health outcomes:

**Four or more ANC visits** denoted that the respondent reported having four or more antenatal care (ANC) visits during the pregnancy leading up to her last live birth in the past five years, based on the older WHO guidelines of adequate care for pregnant women that had been adopted by Liberia and Sierra Leone at the time of the surveys. We do note that 2016 WHO ANC recommendations for a positive pregnancy experience now call for eight or more ANC visits [45].

**Skilled attendance at delivery** indicated whether the respondent reported that her most recent live birth within the past five years was delivered by a doctor, nurse/midwife, or auxiliary midwife.

**Institutional delivery** indicated whether the respondent reported delivering her most recent birth in the past five years in a health facility (public or private).

#### Control variables (independent variables)

**Fig 3:**
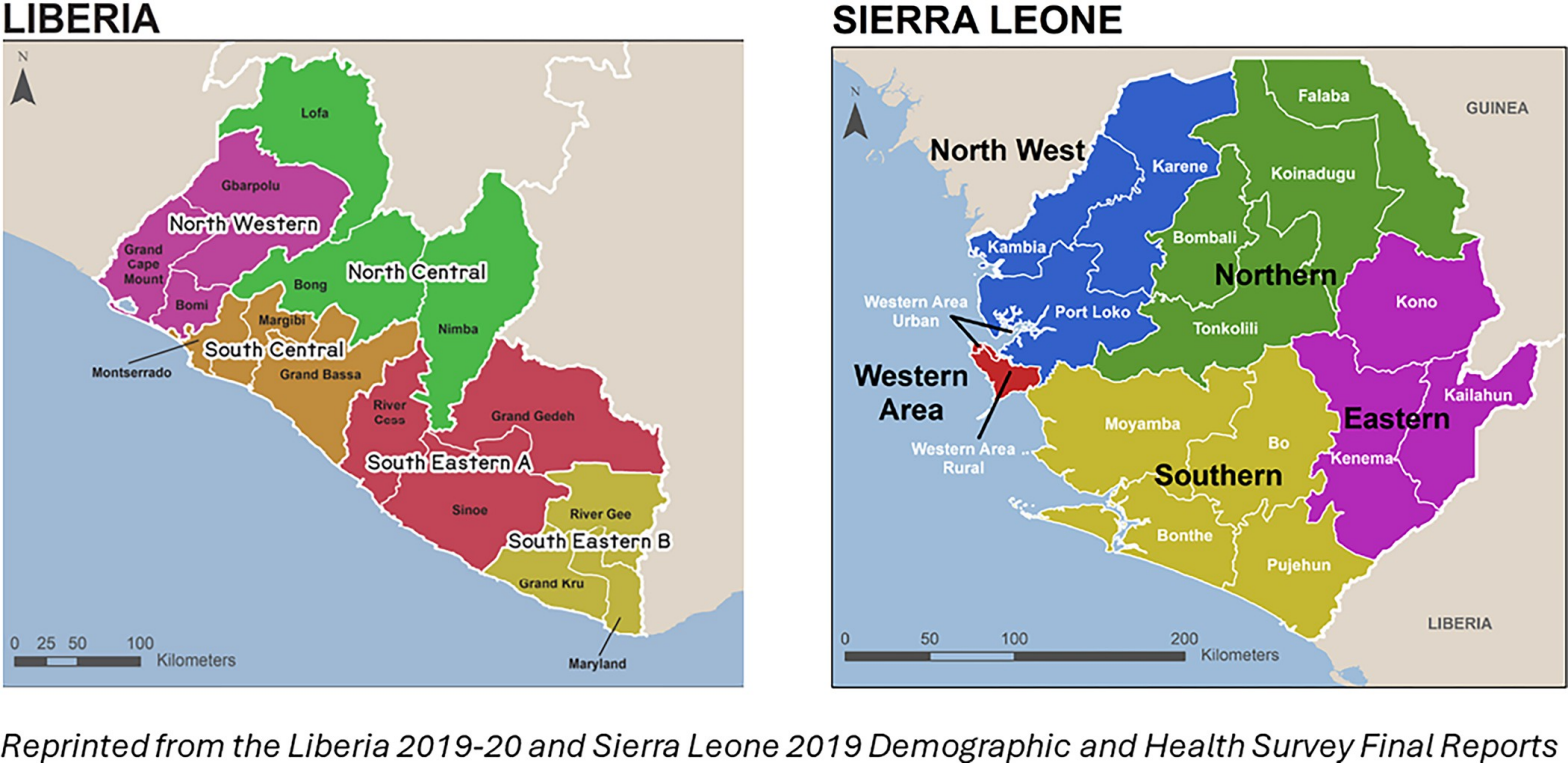
Maps showing DHS regions of Liberia and Sierra Leone.

**Child marriage** was our primary independent variable, defined as first marriage or cohabitation before age 18 years and consisted of three categories: <15, 15–17, and 18 or older (reference group). Other control variables were **household wealth quintile** (poorest, poorer, middle, richer, richest), **woman’s education** (no education, primary, secondary/higher), **religion** (Muslim or non-Muslim), **type of place of residence** (urban or rural), **region** (Liberia: North Western, South Central, Southeastern A, Southeastern B, and North Central; Sierra Leone: Eastern, Northern, North Western, Southern, and Western) (See Fig 3), **number of decisions made alone or jointly with the husband/partner** (decisions about the respondent’s health care, large household purchases, and/or visits to family or relatives), **mobility** (getting permission to go for medical help for self; response options were “not a big problem” or “big problem”), and **ability to negotiate safer sex** (measured by the belief that a wife is justified in asking her husband to use a condom if he has a sexually-transmitted infection (STI); response options were “no/don’t know” or “yes”). We also controlled for **partner’s relative education** (same level, wife more educated, husband/partner more educated) and the **partner age gap** (husband/partner younger than the respondent, husband/partner older by <5 years, 5–9 years older, or 10+ years older). Additional control variables were defined for specific outcomes: number of living sons and the number of family planning message channels to which the respondent was exposed in the past few months (modern contraceptive use) and respondent’s birth order (all maternal health outcomes).

### Statistical methods

Descriptive and multivariable analyses were conducted separately by country, and country differences in outcomes, such as early fertility, low fertility control, and unwanted pregnancies, were analyzed. As the DHS used a two-stage probability sample in each country, the data were weighted to account for differences in probability of selection and interview between respondents in the sample. Multivariable logistic regression models examined the association between child marriage and reproductive and maternal health outcomes in each country. We included interaction terms between child marriage and various equity dimensions (household wealth, urban/rural residence, and region) to examine how overlapping factors of social advantage and disadvantage are associated with reproductive and maternal health outcomes. Regression results were presented as adjusted odds ratios (AOR) with 95% confidence intervals (CI). The statistical significance threshold was set to 0.05. The Hosmer-Lemeshow test was used to evaluate the goodness of fit (GOF) of our logistic regression models.

In Liberia and Sierra Leone, 40 and 47 currently married women, respectively, aged 20–24 had missing data on one or more variables of interest and were dropped from the analyses. Twice as many Liberian women with missing data were married to husbands who were 10 or more years older (57%) compared to their 631 counterparts without missing data (27%; *p* = .010; not shown). The data for Sierra Leone showed that significantly more of the 1,325 women who were retained in the analysis felt they were able to negotiate safer sex (73% versus 69%; *p* = 0.002), had secondary or higher levels of education (45% versus 39%; *p* < 0.001), and resided in rural areas (62% versus 39%; *p* = 0.007) compared to their counterparts with missing values. Fewer Sierra Leonean women retained in the analysis resided in the Western region compared to those with missing values (21% versus 41%; *p* = 0.033). These results are available from the authors upon request and should be considered when interpreting the results. The mean variance inflation factor was estimated at 1.7 for Liberia and 1.5 for Sierra Leone, which implied that the independent variables in our regression models were not highly correlated with one another. Data analysis was conducted in Stata 17 [46].

### Ethics considerations

Permission to access and analyze all data was granted by the DHS Program through its online request platform. All data were fully anonymized before we accessed them. The original survey protocols for Liberia were reviewed and approved by institutional review boards (IRBs) at the University of Liberia Pacific Institute for Research and Evaluation and ICF [6]. The original survey protocols for Sierra Leone were reviewed and approved by IRBs at the Sierra Leone Ethics and Scientific Review Committee and ICF [7].

Ahead of each interview, the interviewer read an informed consent statement out loud to the respondent which included the interview’s purpose, the expected length of the interview, interview procedures, potential risks and benefits, and contact information for someone who could give the respondent more information about the interview. The informed consent statement ensured the respondent that participating was voluntary and that respondents could refuse to answer any of the questions, and that their identity and information would be kept confidential [47]. The respondents gave consent orally and interviewers signed the survey form with the date and time of consent. Interviews were conducted as privately as possible. More information on DHS procedures for informed consent and privacy can be found on their web site [47].

## Results

### Background characteristics of the sample

Table 1 shows the characteristics of the sample by country. In both countries, approximately two in five women first married in middle adolescence (age 15–17), and a lower proportion first married below the age of 15 (12% in Liberia and 16% in Sierra Leone). Statistically significant country differences were seen in education (both women’s education and partners’ relative education), religion, type of place of residence, number of decisions made, mobility, and the partner age gap. Educational attainment among women in both countries was low, with fewer than half of respondents having completed secondary or higher education. A greater percentage of respondents in Sierra Leone said they had never been to school (about one in three, compared to one in four in Liberia). However, while Liberia had a greater percentage of respondents who had the same level of education as their husband/partner than Sierra Leone, no respondent in Liberia indicated she was more educated than her husband/partner, compared to almost one in five respondents in Sierra Leone. A greater percentage of Sierra Leonean respondents reported their husband/partner to be older by 10 or more years compared to Liberian respondents. In terms of religion, the prevalence of women in Sierra Leone who were Muslim was four times higher than that of Liberia (80% vs 20%). More than half (55%) of Liberian respondents lived in an urban residence, compared to about one third (38%) of Sierra Leonean respondents.

**Table 1:** Percent distribution of currently married/cohabiting women aged 20–24 by background characteristics, Liberia (2019–2020) and Sierra Leone (2019).

	Liberia		Sierra Leone	
Background Characteristics	N	%	N	%
Age at first marriage/cohabitation (years)				
<15	92	12.2	230	16.2
15–17	252	39.6	496	37.6
18 and older	287	48.2	599	46.2
Wealth quintile				
Poorest	175	21.5	265	17.6
Poorer	175	22.7	293	21.6
Middle	129	18.3	267	21.1
Richer	94	20.8	289	22.9
Richest	58	16.7	211	16.7
Women’s education ***				
No education	153	24.3	523	37.7
Primary	213	27.6	225	17.2
Secondary/higher	265	48.1	577	45.0
Religion ***				
Muslim (Islam)	104	19.5	1074	80.1
Non-Muslim	527	80.5	251	19.9
Type of place of residence ***				
Urban	234	54.8	441	37.8
Rural	397	45.3	844	62.2
Region				
1	85	7.3	258	20.0
2	154	40.9	323	19.4
3	110	8.4	277	21.7
4	108	5.8	272	17.4
5	174	37.6	195	21.5
Number of decisions made alone or jointly with the husband/partner ***				
0	81	17.0	579	44.8
1	58	8.1	191	13.6
2	82	9.5	131	9.5
3	410	65.4	424	32.1
Mobility *				
Not a big problem	531	84.0	965	75.5
Big problem	100	16.0	360	24.5
Ability to negotiate safer sex				
No/don’t know	139	24.9	371	27.2
Yes	492	75.1	954	72.8
Partner’s relative education *				
Same level	304	51.2	603	43.4
Wife more educated	0	0.0	253	19.2
Husband more educated	327	48.8	469	37.4
Partner’s age gap ***				
Husband/partner younger or older by less than 5 years	220	38.3	324	24.5
Husband older by 5–9 years	222	34.0	429	33.7
Husband older by 10+ years	189	27.7	572	41.8
N	631	100.0	1,325	100.0

% = weighted percentage

Note: For Liberia, region numbers correspond with the following: 1 = North Western, 2 = South Central, 3 = Southeastern A, 4 = Southeastern B, and 5 = North Central. For Sierra Leone, 1 = Eastern, 2 = Northern, 3 = North Western, 4 = Southern, and 5 = Western. Percentages may not add up to 100 due to rounding.

* *p* < 0.05

** *p* < 0.01

*** *p* < 0.001. Significance levels pertain to differences between countries in respondents’ background characteristics, except for region, which was not compared.

Regarding decision-making power, Sierra Leonean respondents fared worse than Liberian respondents. About two thirds (65%) of Liberian respondents said they made or participated in all three decisions (their own health care, large household purchases, and visits to family or relatives), compared to less than one third of Sierra Leonean respondents (32%). Additionally, the proportion of Sierra Leonean respondents who said they did not participate in any of the three decisions was almost two and a half times as high as Liberian respondents (45% vs 17%). In Liberia, a significantly greater percentage of respondents said getting permission to seek medical help would “not be a big problem” (84% in Liberia vs 76% in Sierra Leone). However, in both countries, about three fourths of respondents indicated they do have the ability to negotiate safer sex, measured by the belief that a wife is justified in asking her husband to use a condom if he has an STI.

### Bivariate results

Table 2 shows differentials in selected reproductive and maternal health outcomes among currently married/cohabiting women aged 20–24, by age at first marriage/cohabitation and country. Analyses of maternal health outcomes were restricted to the most recent birth in the past five years. There were significant country differences in the overall prevalence of (a) unwanted/mistimed pregnancy (Liberia: 37%; Sierra Leone: 14%); (b) current modern contraceptive use (Liberia: 27%; Sierra Leone: 18%); and (c) four or more ANC visits (Liberia: 88%; Sierra Leone: 79%).

**Table 2:** Percent of currently married/cohabiting women aged 20–24 with selected reproductive and maternal health outcomes, by age at first marriage/cohabitation, Liberia 2019–2020, and Sierra Leone 2019.

	Age at First Marriage/Cohabitation				
Outcome	<15 years	15–17 years	≥18 years	Total (%)	P- value^a^
**Reproductive Health**					
Early fertility					0.07
Liberia**	28.4	45.7	56.3	48.7	
Sierra Leone***	27.7	36.6	52.9	42.7	
High fertility					0.88
Liberia***	47.9	19.7	9.5	18.2	
Sierra Leone***	38.9	22.7	8.1	18.6	
Low fertility control					0.81
Liberia**	31.0	16.9	10.7	15.6	
Sierra Leone***	26.6	16.3	10.0	15.1	
Unwanted/mistimed pregnancy					<0.001
Liberia	27.7	38.9	36.9	36.6	
Sierra Leone	10.1	14.7	14.5	13.9	
Current modern contraceptive use					0.001
Liberia	32.8	26.7	26.5	27.4	
Sierra Leone	23.6	17.4	16.1	17.8	
**N**					
Liberia	92	252	287	631	
Sierra Leone	230	496	599	1,325	
**Maternal Health** ^**b**^					
≥4 ANC visits					0.002
Liberia	80.8	89.4	88.8	88.0	
Sierra Leone	80.3	80.0	77.5	79.0	
Skilled attendance at delivery					0.74
Liberia *	91.9	82.6	91.9	88.0	
Sierra Leone	90.3	88.6	88.2	88.7	
Institutional delivery					0.96
Liberia	84.2	83.3	88.8	85.8	
Sierra Leone	87.5	85.7	85.0	85.5	
**N**					
Liberia	85	228	218	528	
Sierra Leone	195	445	453	1,093	

Data are weighted.

^a^ Pertains to country differences in the overall prevalence.

^b^ Analysis was restricted to the most recent birth in the past five years.

* *p* < 0.05

** *p* < 0.01

*** *p* < 0.001. In each country, a design-based F-test was used to assess the statistical significance of differences by age at first marriage/cohabitation in reproductive and maternal health outcomes.

In both countries, there was a statistically significant association between the age at first marriage and the prevalence of early fertility, high fertility, and low fertility control. For example, in Liberia, the prevalence of high fertility was five times as high among women who first married before age 15 as among those who did so in adulthood. However, in both countries, the prevalence of early fertility increased steadily with the age at first marriage. For example, in Sierra Leone, early fertility prevalence rates were 28% for age <15, 37% for age 15–17, and 53% for age 18 and older. The only statistically significant age at marriage differential in maternal health outcomes was skilled attendance at delivery. In Liberia only, among women who married between age 15–17, there was about nine percentage points lower prevalence of skilled attendance at delivery compared to those who married under age 15 or as adults.

### Multivariable regression results

Multivariable logistic regression models were run to assess the association between age at first marriage and reproductive and maternal health outcomes, after controlling for socioeconomic factors. All models passed the Hosmer-Lemeshow Goodness of Fit test, except for several outcomes in Liberia: high fertility, current modern contraceptive use, and ≥4 ANC visits. As Table 3 shows, in both countries, child marriage was significantly associated with sharply increased adjusted odds of high fertility, defined as having 3 or more live births, particularly for those married at age <15. However, the model for this outcome in Liberia failed to pass the Goodness of Fit test.

**Table 3:** Adjusted odds ratios (AOR) and 95 percent confidence intervals (CI) from multivariable regression models of the association between age at first marriage/cohabitation and reproductive and maternal health outcomes among currently married/cohabiting women aged 20–24, Liberia 2019–2020 and Sierra Leone 2019.

	< 15 years		15–17 years		
	AOR	95% CI	AOR	95% CI	N
**Reproductive Health**					
Early fertility					
Liberia	0.265**	[0.119, 0.592]	0.610	[0.361, 1.033]	631
GOF	F(9,234) = 0.71, *p* = 0.702				
Sierra Leone	0.347***	[0.236, 0.512]	0.485***	[0.369, 0.638]	1,325
GOF	F(9,465) = 1.19, *p* = 0.297				
High fertility					
Liberia	13.131***	[5.855, 29.450]	2.734**	[1.285, 5.817]	631
GOF	F(9,234) = 3.43, *p* < 0.001				
Sierra Leone	10.863***	[6.436, 18.336]	3.929***	[2.397, 6.439]	1,325
GOF	F(9,465) = 1.53, *p* = 0.136				
Low fertility control (< 24 months)					
Liberia	3.935***	[1.908, 8.115]	1.686	[0.866, 3.282]	631
GOF	F(9,234) = 1.40, *p* = 0.190				
Sierra Leone	3.447***	[2.131, 5.575]	1.728*	[1.139, 2.622]	1,325
GOF	F(9,465) = 1.47, *p* = 0.155				
Unwanted/mistimed pregnancy					
Liberia	0.853	[0.369, 1.974]	1.209	[0.604, 2.418]	631
GOF	F(9,234) = 0.68, *p* = 0.731				
Sierra Leone	0.786	[0.440, 1.405]	1.088	[0.720, 1.644]	1,325
GOF	F(9,465) = 1.07, *p* = 0.380				
Current modern contraceptive use					
Liberia	1.646	[0.754, 3.591]	1.300	[0.730, 2.315]	631
GOF	F(9,234) = 1.97, *p* = 0.043				
Sierra Leone	2.138**	[1.252, 3.650]	1.314	[0.847, 2.038]	1,325
GOF	F(9,465) = 1.10, *p* = 0.363				
**Maternal Health**					
≥4 ANC visits					
Liberia	0.520	[0.151, 4.276]	1.131	[0.430, 2.976]	528
GOF	F(9,220) = 2.17, *p* = 0.025				
Sierra Leone	0.926	[0.541, 1.585]	1.090	[0.756, 1.572]	1,093
GOF	F(9,431) = 0.36, *p* = 0.955				
Skilled attendance at delivery					
Liberia	1.280	[0.447, 3.664]	0.464*	[0.219, 0.987]	528
GOF	F(9,220) = 1.83, *p* = 0.063				
Sierra Leone	1.213	[0.612, 2.401]	1.170	[0.670, 2.040]	1,093
GOF	F(9,431) = 0.46, *p* = 0.903				
Institutional delivery					
Liberia	0.702	[0.284, 1.736]	0.582	[0.259, 1.308]	528
GOF	F(9,220) = 1.69, *p* = 0.092				
Sierra Leone	1.043	[0.567, 1.919]	1.051	[0.653, 1.693]	1,093
GOF	F(9,431) = 0.38, *p* = 0.944				

*** *p* < 0.001

** *p* < 0.01

* *p* < 0.05

GOF = Hosmer-Lemeshow Goodness of Fit test

Reference group: 18+

All regression models controlled for women’s participation in decision making, education, belief in a woman’s right to refuse sex if her husband has a sexually transmitted infection, partners’ relative education, partner age gap, respondent’s age, respondent’s education, household wealth, religion, region, and type of place of residence.

Regression models for modern contraceptive use included additional controls for number of living sons and the number of family planning message channels to which the respondent was exposed. Regression models for maternal health outcomes included additional controls for birth order and pertain to the most recent live birth in the past five years.

Child marriage was also associated with increased adjusted odds of low fertility control, defined as having had at least one birth within 23 months of a previous birth, and the association was especially strong for those married below age 15. For example, in Liberia, the AOR of low fertility control (<24 months) was almost four times as high among women who married before the age of 15, (*AOR* = 3.94, 95% CI [2.13, 5.58]) compared to women who married as adults (age 18 or older). There was not a significant difference in the odds of low fertility control between Liberian women who married at age 15–17 and their counterparts who married in adulthood. The opposite was true for this same marriage age group in Sierra Leone, *AOR* = 1.73, [1.14, 2.62]. We also analyzed low fertility control considering WHO minimum birth-to-birth interval of 33 months and found Sierra Leonean women who were married <15 had almost six times the adjusted odds of this outcome (*AOR* = 5.58, [3.819, 8.16]), and those married in middle adolescence had almost three times the adjusted odds of this outcome, *AOR* = 2.81, [2.03, 3.89]. The analysis also showed Liberian women married below 15 had higher odds of this outcome, but the model failed to pass the Goodness of Fit test, and the information is available upon request.

In Sierra Leone, women who were married below the age of 18 had significantly lower odds of experiencing early fertility compared to those who married in adulthood, age <15: *AOR* = 0.35, [0.24, 0.51]; age 15–17: *AOR* = 0.49, [0.37, 0.64]. In Liberia, early fertility was significant only for those married below the age of 15, *AOR* = 0.27, [0.12, 0.59].

In Sierra Leone only, women who were married under the age of 15 had significantly higher odds of current modern contraceptive use compared to those who married as adults. Age at first marriage was not significantly associated with unwanted/mistimed pregnancy, four or more ANC visits, or institutional delivery in either country. In Liberia, but not in Sierra Leone, child marriage was significantly associated with lower odds of skilled attendance at delivery, but only for women who were married in middle adolescence, *AOR* = 0.46, [0.22, 0.99]. The full regression models are shown in S1 and S2 Appendices for Liberia and in S3 and S4 Appendices for Sierra Leone.

Country differences in the adjusted odds of reproductive and maternal health outcomes of interest were examined by merging the data sets for both countries. A country variable was included in the regression models, with Liberia as the reference category. The results of these models indicated that the odds of having an unintended pregnancy (*AOR* = 0.39, [0.22, 0.66], *p* = 0.001) and four or more ANC visits (*AOR* = 0.37, [0.18, 0.77], *p* = 0.008) were significantly lower in Sierra Leone than in Liberia (data available upon request).

We also explored whether age at first marriage/cohabitation intersected with various equity dimensions (household wealth, urban/rural residence, and region) to influence reproductive and maternal health outcomes. In both countries, none of the interaction terms between age at first marriage and household wealth were statistically significant. This meant that the association between age at first marriage and each outcome did not depend on the level of household wealth (not shown). We also found that the association between age at first marriage/cohabitation and Liberian women’s odds of having skilled attendance at delivery was moderated by type of place of residence (age 15–17: *AOR* = 1.34, [0.29, 6.84]; age < 15: *AOR* = 0.03, [0.00, 0.47], *p* = 0.013). No other interaction terms between age at first marriage/cohabitation and type of place of residence were statistically significant in Liberia or Sierra Leone.

Regression results shown in Table 3 suggested that age at first marriage was more strongly associated with reproductive health outcomes than with maternal health outcomes. However, as indicated in Table 4, in both countries, the association between age at marriage and maternal health outcomes depended on region. In Liberia, women who married at age 15–17 had significantly lower odds of skilled attendance at delivery (*AOR* = 0.02, [0.00, 0.36]) and institutional delivery (*AOR* = 0.01, [0.00, 0.19]) if they lived in the North Central region versus the North Western region. The AORs associated with both interaction terms were statistically significant at the one percent level. Sierra Leonean women who married before age 15 had significantly lower odds of having four or more ANC visits (*AOR* = 0.17, [0.04, 0.76]) and institutional delivery (*AOR* = 0.08, [0.01, 0.98]) if they lived in the North Western region than in the Eastern region. In addition, Sierra Leonean women had significantly lower odds of institutional delivery if they lived in the Western region than in the Eastern region. In Sierra Leone, the interaction between age at marriage and region was statistically significant at the five percent level.

**Table 4:** Adjusted odds ratios (AOR) and 95 percent confidence intervals (CI) from multivariable regression models of the association between age at first marriage/cohabitation and maternal health outcomes among women aged 20–24, with interactions between age at first marriage/cohabitation and region of residence, Liberia 2019–2020, and Sierra Leone 2019.

	Four or More ANC Visits		Skilled Attendance at Delivery		Institutional Delivery	
	AOR	95% CI	AOR	95% CI	AOR	95% CI
**LIBERIA **						
**Age at first marriage/ cohabitation) **						
18 and older	1.000		1.000		1.000	
15–17	1.411	[0.226, 8.812]	2.061	[0.433, 9.803]	2.848	[0.699, 11.607]
<15	0.681	[0.091, 5.111]	0.228	[0.016, 3.243]	0.293	[0.025, 3.379]
**Region **						
North Western	1.000					
South Central	1.022	[0.204, 5.135]	0.594	[0.080, 4.391]	0.480	[0.091, 2.546]
South Eastern A	0.691	[0.122, 3.933]	2.674	[0.389, 18.394]	0.978	[0.179, 5.344]
South Eastern B	0.808	[0.122, 3.933]	1.137	[0.194, 6.661]	1.029	[0.229, 4.623]
North Central	2.337	[0.172, 3.795]	33.589**	[2.569, 439.208]	33.584**	[3.020, 373.430]
**Interactions **						
15–17 x South Central	0.887	[0.071, 11.144]	0.256	[0.029, 2.247]	0.276	[0.034, 2.277]
15–17 x South Eastern A	0.893	[0.095, 8.388]	0.545	[0.064, 4.637]	0.470	[0.072, 3.071]
15–17 x South Eastern B	0.771	[0.099, 6.031]	0.489	[0 .060, 3.966]	0.352	[0.054, 2.292]
15–17 x North Central	0.652	[0.056, 7.535]	0.024**	[0.002, 0.364]	0.014**	[0.001, 0.186]
<15 x South Central	0.369	[0.034, 3.961]	8.099	[0.309, 212.432]	1.710	[0.096, 30.458]
<15 x South Eastern A	3.505	[0.252, 48.829]	3.867	[0.204, 73.241]	3.054	[0.223, 41.876]
<15 x South Eastern B	3.009	[0.157, 57.809]	4.122	[0.166, 102.594]	3.437	[0.197, 60.000]
<15 x North Central	0.932	[0.070, 12.334]	2.204	[0.040, 121.460]	1.265	[0.027, 58.348]
**N **	**528 **		**528 **		**528 **	
**SIERRA LEONE **						
**Age at first marriage/ cohabitation) **						
18 and older	1.000		1.000		1.000	
15–17	0.925	[0.386, 2.218]	1.377	[0.275, 6.888]	1.228	[0.329, 4.589]
<15	1.887	[0.573, 6.213]	2.365	[0.284, 19.663]	8.829	[0.849, 91.766]
**Region **						
Eastern	1.000		1.000		1.000	
Northern	1.939	[0.729, 5.156]	0.555	[0.135, 2.289]	0.526	[0.173, 1.603]
North Western	0.827	[0.387, 1.770]	0.207*	[0.058, 0.743]	0.261*	[0.089, 0.762]
Southern	0.460*	[0.219, 0.968]	1.087	[0.245, 4.826]	1.354	[0.375, 4.889]
Western	0.276**	[0.115, 0.661]	0.406	[0.081, 2.026]	0.304	[0.085, 1.083]
**Interactions **						
15–17 x Northern	0.485	[0.132, 1.783]	0.735	[0.113, 4.771]	0.580	[0.125, 2.693]
15–17 x North Western	1.300	[0.453, 3.732]	1.253	[0.216, 7.273]	1.166	[0.270, 5.029]
15–17 x Southern	1.183	[0.403, 3.471]	0.367	[0.046, 2.929]	0.495	[0.076, 3.214]
15–17 x Western	1.521	[0.444, 5.212]	0.759	[0.078, 7.371]	1.107	[0.200, 6.144]
<15 x Northern	0.486	[0.088, 2.694]	0.439	[0.039, 4.897]	0.117	[0.010, 1.403]
<15 x North Western	0.170*	[0.038, 0.764]	0.417	[0.041, 4.262]	0.083*	[0.007, 0.983]
<15 x Southern	0.436	[0.099, 1.909]	1.526	[0.066, 35.044]	0.374	[0.014, 9.762]
<15 x Western	0.683	[0.117, 3.985]	0.232	[0.010, 5.688]	0.043*	[0.003, 0.674]
**N **	**1,093 **		**1,093 **		**1,093 **	

*** *p* < 0.001

** *p* < 0.01

* *p* < 0.05

All regression models controlled for women’s participation in decision making, women’s education, belief in a woman’s right to refuse sex if her husband has a sexually transmitted infection, partners’ relative education, partner age gap, respondent’s age, household wealth, religion, type of place of residence. Women’s participation in decision making, education, partners’ relative education, partner age gap, and household wealth were defined as continuous variables in these regression models.

Regression models for modern contraceptive use include additional controls for number of living sons and the number of family planning message channels to which the respondent was exposed. Regressions models for maternal health outcomes include additional controls for birth order and pertain to the most recent live birth in the past five years.

**Fig 4:**
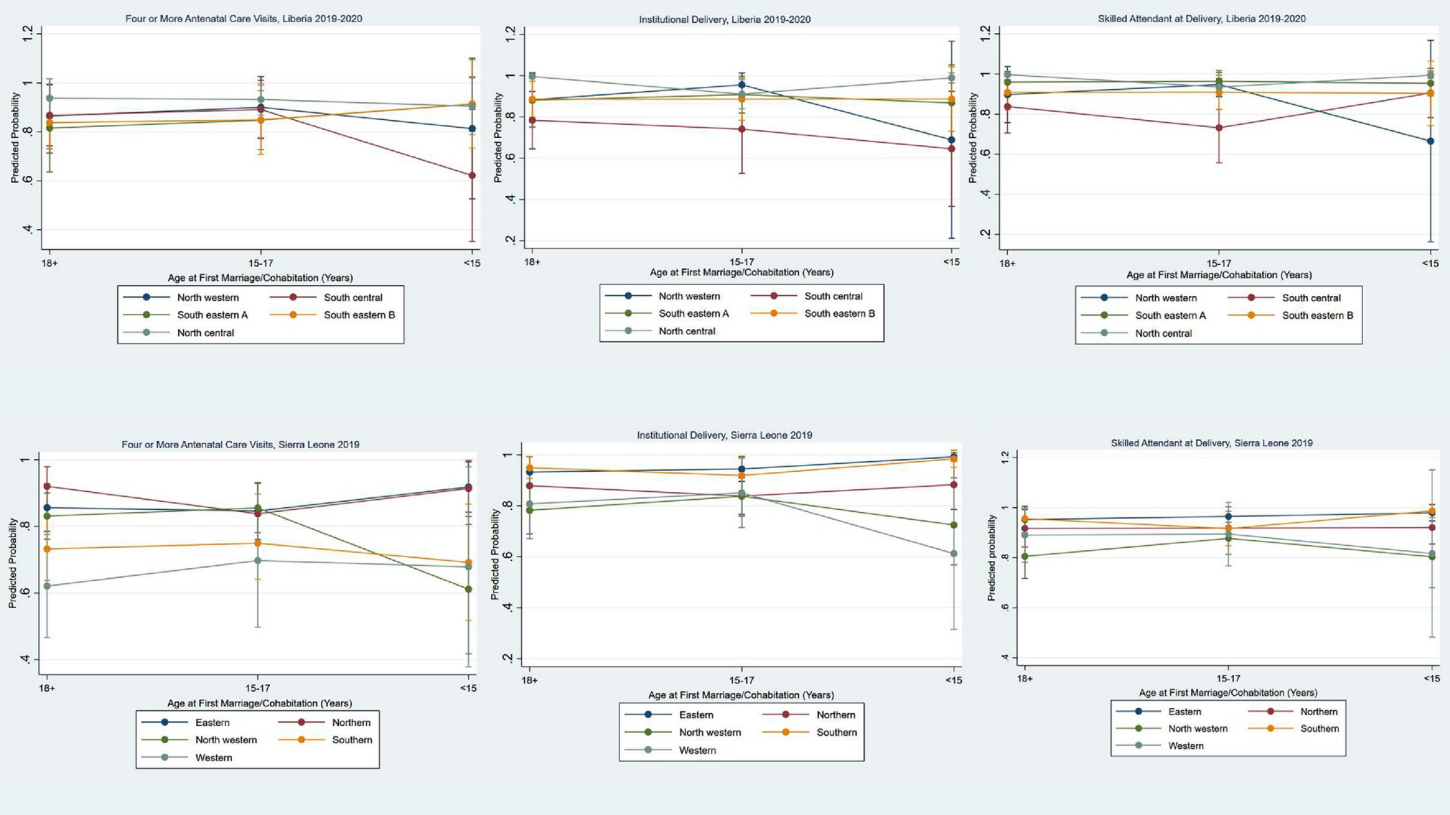
Adjusted predictions of the interaction between age at first marriage/cohabitation and geographic region with 95% confidence intervals for selected maternal health outcomes, Liberia 2019–2020, and Sierra Leone 2019.

To facilitate interpretation of the interactions, we calculated predictive margins for each maternal health outcome based on age at first marriage and geographic region of residence, after adjustment for the sample design. Margins were estimated at the means of covariates included in the regression models presented in Table 4. Fig 4 presents the margin plots for each country and outcome. Regarding four or more ANC visits, interaction effects could be discerned in Sierra Leone, where the predicted probability was lower among women in the North Western region who first married before age 15 (0.61) as compared to those in the same region who married at age 15–17 (0.85) or age 18 or older (0.83). In contrast, no clear pattern in the probability of four or more ANC visits was observed in the other regions, except for the Western region, where the predicted probability was lower among women who married in adulthood (0.62) than among those who married in childhood (age 15–17: 0.70; age < 15: 0.68).

In Liberia, there was evidence of interaction effects in the probability of skilled attendance at delivery. In the North Western region, the age at first marriage differences in the probability was striking (about 28 percentage points between first marriage before age of 15 (0.67) and at age 15–17 (0.95)). The North Western region also showed the steepest increase in the probability of institutional delivery between women who married before age 15 and those who married at age 15–17. Only one region, South Central, showed a gradual increase in the probability of institutional delivery with an increase in the age at first marriage/cohabitation.

In both the Western and North Western regions of Sierra Leone, the predicted probability of institutional delivery increased sharply from marriage before age 15 to marriage at ages 15–17. In contrast, we saw a decreasing probability of institutional delivery between women who first married at ages 15–17 and those who married in adulthood. In other regions of the country, the predicted probability of institutional delivery remained almost constant across age at marriage categories.

## Discussion

This study examined the association between child marriage and selected reproductive and maternal health outcomes in Liberia and Sierra Leone. Our hypotheses stated that there were country differences in the association between child marriage and reproductive and maternal health outcomes, that child marriage intersected with social disadvantages, and that child marriage influenced maternal and reproductive health outcomes. The key findings indicated that, in both countries, child marriage was a risk factor for high fertility and low fertility control, and region of residence moderated the associations between child marriage and some maternal health outcomes, a possible reflection of access to quality healthcare services.

Our findings showed a significant association between age at first marriage and increased odds of high fertility and low fertility control which aligned with prior studies [14, 16, 18, 48]. Previous literature suggested several mechanisms, such as low decision-making power among girls, lower knowledge of modern contraceptives, and social norms prioritizing large families [16], may explain the positive association between child marriage and high fertility as well as low fertility control. The current study’s finding that child marriage at ages 15–17 was significantly associated with lower odds of skilled attendance at delivery in Liberia was also in line with previous research [16, 48, 49], as was the negative association between child marriage and early fertility [18]. The association of child marriage with lower odds of early fertility could point to social norms around childbearing, frequency of sex, and/or low fecundity at younger ages. As other authors have suggested [16, 50], low frequency of intercourse could be explained by traditions around marriage consummation, living arrangements, and/or arranged or forced marriages.

However, in contrast with our study, Yaya et al. [18] found a significantly lower odds of low fertility control (termed “rapid repeat of childbirth” in the study) among women who were married below the age of 18. The study, however, combined DHS data from 34 sub-Saharan African countries, with varying demographic, cultural, and educational conditions, and a nine-year gap between some of the surveys analyzed.

Mixed regional results on the statistical significance of the negative association between first marrying/cohabiting at 15–17 and the odds of having skilled attendance at delivery were also found in the multi-country study by Godha et al. [16]. Perhaps the results are explained by societal recognition of greater risks during delivery among very young mothers, or other sociocultural ideas around childbirth. In other words, perhaps extra caution is taken when a very young mother is in labor.

While we found no significant association between child marriage and unwanted/mistimed pregnancies, according to a 2022 systematic review [51], previous research based on DHS surveys show mixed results for this outcome. While one study of 34 African nations reported a protective effect of child marriage against unwanted/mistimed pregnancies [18], other studies from South Asia found child marriage increased odds of this health outcome [16, 52], except for a handful of studies involving Bangladesh that found mixed results for this association [16, 17, 53]. It is possible that the women interviewed in sub-Saharan Africa hold certain beliefs, such as the ideal of a large family size that is prevalent across the region, making them less likely to openly acknowledge an unwanted/mistimed pregnancy during a survey. Alternatively, unwanted/mistimed pregnancy may be subject to “post facto rationalization” as Godha et al. suggested [16].

One strength of this study is that it built on previous research looking at reproductive and maternal health outcomes because it specifically compared Liberia and Sierra Leone. Another strength of this study is that by identifying the country and regional differences, recommendations can be made to address the obstacles women who were married in adolescence face in Liberia and Sierra Leone in order to improve reproductive and maternal health outcomes.

In our study, child marriage was also not significantly associated with number of ANC visits, when controlling for other variables. This was in agreement with another recent study, also based on DHS surveys of 20 sub-Saharan African nations, which included Liberia and Sierra Leone [12]. When examining pooled data of these 20 nations, the study found a significantly lower proportion of child brides had four or more ANC visits compared to adult brides. However, the association between child marriage and four or more ANC visits for Liberia and Sierra Leone individually was not statistically significant. Another 2022 systematic review [54] highlighted a few studies from South Asia that found child brides had fewer ANC visits compared to women married as adults [16, 55, 56].

Additionally, when controlling for other variables, our finding that child marriage had no significant association with institutional delivery is in contrast with previous research that has found child brides from South Asia and sub-Saharan Africa had lower odds of delivering in a health facility [12, 16, 55, 57, 58]. However, in our study there were significant interactions between region of residence and lower odds of institutional delivery, indicating that the effect of child marriage on institutional delivery may depend on which region was studied. Furthermore, access to health facilities and cultural norms may also vary by region, and this calls for more research to explore the underlying mechanisms behind this variation.

Unfortunately, few studies have examined how child marriage combines with women’s social characteristics to create reproductive and maternal health risks or protections, limiting our ability to relate relevant findings to previously published work. In this study, an unexpected finding was that in Liberia, there was not a significant difference in the odds of low fertility control between women who married in childhood and women who married in adulthood (after age 18). It was also notable that in Sierra Leone, women who were married before age 15 had significantly higher odds of using modern contraception than those married at age 18 or older. This finding may indicate that women who married in adulthood faced greater social pressure to bear children once married due to strong cultural values that link marital fertility with marital stability, whereas women who were married younger tried to control how many children they would have. A question raised was: Did their partners know that the women were using contraception? This question is important because it addresses whether decisions were being made together and how much autonomy women had when it came to reproductive health.

Our study contributes significantly to previous research because it showed how variables, such as region and age at first marriage, can have negative consequences for reproductive and maternal health outcomes. Although this study investigated country differences for reproductive and maternal health outcomes between Liberia and Sierra Leone, there were many unanswered questions such as, were women able to choose for themselves whether to get married, at what age, and to whom? This question can lead to future research examining women’s autonomy in marriage and childbearing decisions.

### Study limitations

Although the DHS is nationally-representative, the data are subject to recall and reporting bias. In small and remote areas, the sample sizes are sometimes not large enough to capture the full picture. Our analysis of high fertility was limited by the small number of women aged 20–24 who first married before age 15 and who reported three or more live births at the time of the interview, leading to wide confidence intervals for the adjusted odds ratios. As the data were cross-sectional, temporal ordering of our variables could not be established with a high level of certainty.

Despite these limitations, by highlighting significant regional differences, we have helped to lay the groundwork for future community-based research looking into why women who married at certain ages in certain regions have higher or lower odds of certain reproductive and maternal health outcomes. To our knowledge, this was the first study that compared child marriage and reproductive and maternal health outcomes in Liberia and Sierra Leone. It was also the first to discover major regional differences within these countries in the interaction between age at first marriage/cohabitation and maternal health outcomes.

### Program and policy implications

Research has shown that when girls marry later in life, they have fewer children and better health outcomes throughout the life course. However, child marriage persists in Liberia and Sierra Leone, in part, because policies defining the legal age of marriage are often not enforced, and in Sierra Leone’s plural legal system, statutory law based on international agreements is in tension with customary laws permitting child marriage. Policy advocates may want to consider this study’s findings when addressing contradictory laws that do not fully protect the health and wellbeing of adolescent girls. Governments could implement new policies that incentivize families to keep their children in school longer, such as financial assistance or scholarships as well as encouraging women to have fewer children.

Governments should improve health systems to better respond to the needs and priorities of women and girls, and they may want to consider this study’s findings that highlight the special vulnerability of child brides to negative maternal and reproductive health outcomes when allocating resources. One program-centered strategic objective suggested by the Trends in maternal mortality 2000 to 2020 report, co-authored by WHO, UNICEF, and others, is to address the inequities that affect access to, and quality of, sexual, reproductive, maternal, and newborn health care [59]. In line with this objective, the Liberian and Sierra Leonean governments should consider improving infrastructure in areas with lower rates of institutional delivery and antenatal care visits and increasing the availability of skilled birth attendants in these regions to increase equitable, evidence-based care for this vulnerable population. There also may be a call for qualitative research examining cultural traditions regarding where women should deliver their first birth that may influence these regional disparities.

Negative maternal and reproductive health outcomes can continue to be combatted through programmatic interventions focused on a proactive approach to family planning. Family planning programs in both nations should seek to provide education before and throughout the reproductive years on the importance of contraceptive use and women’s decision-making power. Programs should also seek to discover the mechanisms behind interactions between age at first marriage and region of residence; for example, why women who were married between ages 15–17 and live in the Libera’s South central region have lower predicted probability of skilled attendance at delivery (compared to women married below age 15 or at age 18+). Public health campaigns may need to educate families and mothers-to-be on the importance of skilled attendance at delivery for all births—not just adolescent girls’ births. Programs should also stress the importance of WHO-aligned recommendations for antenatal care visits and institutional delivery for all women and girls.

## Conclusion

Child marriage in Liberia and Sierra Leone remains a pressing problem. As this study demonstrated, the practice is clearly associated with negative maternal and reproductive health outcomes. Distinguishing first marriages before or during early adolescence from first marriages occurring in mid-adolescence and examining interactions between age at first marriage and region have provided valuable insight for further exploration.

## Supporting information

S1 Appendix Adjusted odds ratios and 95% confidence intervals for full regression models of the association between child marriage and reproductive health outcomes, currently married women aged 20–24, Liberia 2019–2020. (DOCX)

S2 Appendix Adjusted odds ratios and 95% confidence intervals for full regression models of the association between child marriage and maternal health outcomes, currently married women aged 20–24, Liberia 2019–2020. (DOCX)

S3 Appendix Adjusted odds ratios and 95% confidence intervals for full regression models of the association between child marriage and reproductive health outcomes, currently married women aged 20–24, Sierra Leone 2019. (DOCX)

S4 Appendix Adjusted odds ratios and 95% confidence intervals for full regression models of the association between child marriage and maternal health outcomes, currently married women aged 20–24, Sierra Leone 2019. (DOCX)

S5 Appendix Adjusted odds ratios and 95% confidence intervals for the association between child marriage and reproductive health outcomes after adjusting for other factors, currently married women aged 20–24, Sierra Leone 2019 and Liberia 2019–2020 combined. (DOCX)

S6 Appendix Adjusted odds ratios and 95% confidence intervals for the association between child marriage and maternal health outcomes after adjusting for other factors, currently married women aged 20–24, Sierra Leone 2019 and Liberia 2019–2020 combined. (DOCX)

S7 Appendix Adjusted odds ratios and confidence intervals from regressions of the association of child marriage with reproductive and maternal health outcomes, currently married women aged 20–24, Sierra Leone 2019 and Liberia 2019–2020. (DOCX)

S1 File (DOCX)

## References

[1] United Nations Office of the High Commissioner for Human Rights. Child and forced marriage, including in humanitarian settings: OHCHR and women’s human rights and gender equality. n.d. Available from: https://www.ohchr.org/en/women/child-and-forced-marriage-including-humanitarian-settings.

[2] United Nations. The Sustainable Development Goals Report 2022. 2022.

[3] UNICEF. Child marriage: Child marriage threatens the lives, well-being and futures of girls around the world. 2023. Available from: https://www.unicef.org/protection/child-marriage.

[4] Gastón CM, Misunas C, Cappa C. Child marriage among boys: a global overview of available data. Vulnerable Children and Youth Studies. 2019;14(3):219–28.

[5] United Nations Children’s Fund. Child Marriage in West and Central Africa: A statistical overview and reflections on ending the practice. New York; 2022.

[6] Liberia Institute of Statistics and Geo-Information Services (LISGIS); Ministry of Health [Liberia]; ICF. Liberia Demographic and Health Survey 2019–20. Monrovia, Liberia and Rockville, Maryland, USA: Liberia Institute of Statistics and Geo-Information Services (LISGIS), Ministry of Health, and ICF; 2021.

[7] Statistics Sierra Leone (Stats SL), ICF. Sierra Leone Demographic and Health Survey 2019. Freetown, Sierra Leone; 2020.

[8] Sagalova V, Nanama S, Zagre NM, Vollmer S. Long-term consequences of early marriage and maternity in West and Central Africa: Wealth, education, and fertility. J Glob Health. 2021;11:13004. doi: 10.7189/jogh.11.13004 34484711 PMC8397277

[9] Delprato M, Akyeampong K, Sabates R, Hernandez-Fernandez J. On the impact of early marriage on schooling outcomes in Sub-Saharan Africa and South West Asia. International Journal of Educational Development. 2015;44:42–55.

[10] Adhikari R. Child Marriage and Physical Violence: Results from a Nationally Representative Study in Nepal. Journal of Health Promotion. 2018;6:49–59.

[11] Hayes BE, Protas ME. Child Marriage and Intimate Partner Violence: An Examination of Individual, Community, and National Factors. Journal of Interpersonal Violence. 2022;37(21–22):NP19664–NP87. doi: 10.1177/08862605211042602 34476987

[12] Adedini SA, Abatan SM, Ogunsakin AD, Alex-Ojei CA, Babalola BI, Shittu SB, et al. Comparing the timeliness and adequacy of antenatal care uptake between women who married as child brides and adult brides in 20 sub-Saharan African countries. PLOS ONE. 2022;17(1):e0262688. doi: 10.1371/journal.pone.0262688 35025949 PMC8758032

[13] Ahinkorah BO, Budu E, Seidu AA, Bolarinwa OA, Agbaglo E, Adu C, et al. Girl child marriage and its association with maternal healthcare services utilization in sub-Saharan Africa. BMC Health Serv Res. 2022;22(1):777. 35698223 10.1186/s12913-022-08117-9 PMC9195447

[14] Elnakib S, Elsallab M, Wanis MA, Elshiwy S, Krishnapalan NP, Naja NA. Understanding the impacts of child marriage on the health and well-being of adolescent girls and young women residing in urban areas in Egypt. Reproductive Health. 2022;19(1):8. doi: 10.1186/s12978-021-01315-4 35033114 PMC8761304

[15] Amoadu M, Hagan D, Ansah EW. Adverse obstetric and neonatal outcomes of adolescent pregnancies in Africa: a scoping review. BMC Pregnancy Childbirth. 2022;22(1):598. doi: 10.1186/s12884-022-04821-w 35896998 PMC9327294

[16] Godha D, Hotchkiss DR, Gage AJ. Association Between Child Marriage and Reproductive Health Outcomes and Service Utilization: A Multi-Country Study From South Asia. Journal of adolescent health. 2013;52(5):552–8. doi: 10.1016/j.jadohealth.2013.01.021 23608719

[17] Kamal SMM. Decline in Child Marriage and Changes in Its Effect on Reproductive Outcomes in Bangladesh. Journal of Health, Population and Nutrition. 2012;30(3). doi: 10.3329/jhpn.v30i3.12296 23082634 PMC3489948

[18] Yaya S, Odusina EK, Bishwajit G. Prevalence of child marriage and its impact on fertility outcomes in 34 sub-Saharan African countries. BMC Int Health Hum Rights. 2019;19(1):33. doi: 10.1186/s12914-019-0219-1 31856810 PMC6924035

[19] Neal S, Mahendra S, Bose K, Camacho AV, Mathai M, Nove A, et al. The causes of maternal mortality in adolescents in low and middle income countries: a systematic review of the literature. BMC Pregnancy and Childbirth. 2016;16(1). doi: 10.1186/s12884-016-1120-8 27836005 PMC5106816

[20] Ganchimeg T, Ota E, Morisaki N, Laopaiboon M, Lumbiganon P, Zhang J, et al. Pregnancy and childbirth outcomes among adolescent mothers: a World Health Organization multicountry study. BJOG: An International Journal of Obstetrics & Gynaecology. 2014;121(s1):40–8. doi: 10.1111/1471-0528.12630 24641534

[21] Trends in maternal mortality 2000 to 2020 [Internet]. The World Bank. 2023. Available from: https://data.worldbank.org/indicator/SH.MMR.RISK?end=2020&start=2000&view=map.

[22] Statistics LIo, Geo-Information Services—LISGIS, Minsitry of Health—MOH, ICF. Liberia Demographic and Health Survey 2019–20. Monrovia, Liberia: LISGIS/MOH/ICF; 2021.

[23] Sierra Leone Visitor Information. About Sierra Leone: History. United Nations Integrated Peacebuilding Office in Sierra Leone. n.d. Available from: https://unipsil.unmissions.org/about-sierra-leone-history.

[24] Dennis P. A Brief History of Liberia. The International Center for Transitional Justice; 2006.

[25] United Nations Development Programme. Human Development Insights. 2022. Available from: https://hdr.undp.org/data-center/country-insights#/ranks.

[26] The World Factbook. Sierra Leone. Central Intelligence Agency. 2024. Available from: https://www.cia.gov/the-world-factbook/countries/sierra-leone/#people-and-society.

[27] The World Factbook. Liberia. Central Intelligence Agency. 2024. Available from: https://www.cia.gov/the-world-factbook/countries/liberia/.

[28] Amberg F, Chansa C, Niangaly H, Sankoh O, De Allegri M. Examining the relationship between armed conflict and coverage of maternal and child health services in 35 countries in sub-Saharan Africa: a geospatial analysis. The Lancet Global Health. 2023;11(6):e843–e53. doi: 10.1016/S2214-109X(23)00152-3 37202021

[29] Chi PC, Bulage P, Urdal H, Sundby J. Perceptions of the effects of armed conflict on maternal and reproductive health services and outcomes in Burundi and Northern Uganda: a qualitative study. BMC International Health and Human Rights. 2015;15(1). 10.1186/s12914-015-0045-z PMC4392810 25884930

[30] DiGiuseppe M, Haer R. The wedding bells of war: The influence of armed conflict on child marriages in West Africa. Journal of Peace Research. 2023;60(3):474–88.

[31] United Nations Development Programme, The World Bank, European Union, African Development Bank. Recovering from the Ebola Crisis. 2015.

[32] Korkoya DT Jr, Wreh FF. EBOLA IMPACT REVEALED: An Assessment of the Differing Impact of the Outbreak on Women and Men in Liberia. 2015.

[33] Lunghi I, Babington-Ashaye A, Vassalli J-D, Heller Y, Michaud P-A, Wernli D, et al. The impact of the Ebola epidemics on children’s rights: a scoping review. Global Health Action. 2022;15(1). doi: 10.1080/16549716.2022.2061240 35506948 PMC9090402

[34] United Nations Children’s Fund. Child marriage and the law: technical note for the global programme to end child marriage. 2020.

[35] The Registration of Customary Marriage and Divorce Act, 2009, (2009).

[36] The Child Rights Act, 2007, (2007).

[37] Dağ H, Yetim A, Ketenci Altıkardeşler Ö, Hançerli Törün S. A Child Abuse: Marriage at Childhood Age. Turk Arch Pediatr. 2021;56(6):548–52. doi: 10.5152/TurkArchPediatr.2021.21093 35110052 PMC8849463

[38] Psaki SR, Melnikas AJ, Haque E, Saul G, Misunas C, Patel SK, et al. What Are the Drivers of Child Marriage? A Conceptual Framework to Guide Policies and Programs. Journal of Adolescent Health. 2021;69(6, Supplement):S13–S22. 10.1016/j.jadohealth.2021.09.001 34809895

[39] United Nations Children’s Fund, United Nations Population Fund. Key drivers of the changing prevalence of child marriage in three countries in South Asia. UNICEF ROSA/UNFPA APRO; 2018.

[40] United Nations Children’s Fund. Ending Child Marriage: Progress and prospects. New York2014.

[41] Tenkorang EY. Explaining the links between child marriage and intimate partner violence: Evidence from Ghana. Child Abuse & Neglect. 2019;89:48–57. doi: 10.1016/j.chiabu.2019.01.004 30622050

[42] Norton M, Chandra-Mouli V, Lane C. Interventions for Preventing Unintended, Rapid Repeat Pregnancy Among Adolescents: A Review of the Evidence and Lessons From High-Quality Evaluations. Global Health: Science and Practice. 2017;5(4):547–70. doi: 10.9745/GHSP-D-17-00131 29284694 PMC5752603

[43] World Health Organization. Technical Consultation and Scientific Review of Birth Spacing. 2005.

[44] Guide to DHS Statistics DHS-8. Current Use of Modern Contraceptive Methods. Demographic and Health Surveys. n.d. Available from: https://www.dhsprogram.com/data/Guide-to-DHS-Statistics/Current_Use_of_Contraceptive_Methods.htm.

[45] World Health Organization. WHO recommendations on antenatal care for a positive pregnancy experience. Geneva, Switzerland 2016. 28079998

[46] StataCorp. Stata Statistical Software: Release 17. College Station, Texas: StataCorp LLC; 2021.

[47] Demographic and Health Surveys. Protecting the Privacy of DHS Survey Respondents. Available from: https://dhsprogram.com/Methodology/Protecting-the-Privacy-of-DHS-Survey-Respondents.cfm.

[48] Nasrullah M, Zakar R, Krämer A. Effect of Child Marriage on Use of Maternal Health Care Services in Pakistan. Obstetrics & Gynecology. 2013;122(3):517–24. doi: 10.1097/AOG.0b013e31829b5294 23921855

[49] Raj A, Boehmer U. Girl child marriage and its association with national rates of HIV, maternal health, and infant mortality across 97 countries. Violence Against Women. 2013;19(4):536–51. doi: 10.1177/1077801213487747 23698937

[50] Dommaraju P, DHS M. Marriage age and fertility dynamics in India: Macro International Incorporated; 2008.

[51] Fan S, Koski A. The health consequences of child marriage: a systematic review of the evidence. BMC Public Health. 2022;22(1). 10.1186/s12889-022-12707-x PMC8845223 35164724

[52] Nasrullah M, Muazzam S, Bhutta ZA, Raj A. Girl Child Marriage and Its Effect on Fertility in Pakistan: Findings from Pakistan Demographic and Health Survey, 2006–2007. Maternal and Child Health Journal. 2014;18(3):534–43. doi: 10.1007/s10995-013-1269-y 23580067

[53] Kamal SMM. Domestic Violence, Unwanted Pregnancy and Pregnancy Termination among Urban Women of Bangladesh. Journal of Family and Reproductive Health. 2013;7(1):11–22. 24971097 PMC4064740

[54] Subramanee SD, Agho K, Lakshmi J, Huda MN, Joshi R, Akombi-Inyang B. Child Marriage in South Asia: A Systematic Review. International Journal of Environmental Research and Public Health. 2022;19(22):15138. doi: 10.3390/ijerph192215138 36429857 PMC9691026

[55] Paul P, Chouhan P. Association between child marriage and utilization of maternal health care services in India: Evidence from a nationally representative cross-sectional survey. Midwifery. 2019;75:66–71. doi: 10.1016/j.midw.2019.04.007 31009933

[56] Sekine K, Carter DJ. The effect of child marriage on the utilization of maternal health care in Nepal: A cross-sectional analysis of Demographic and Health Survey 2016. PLOS ONE. 2019;14(9):e0222643. doi: 10.1371/journal.pone.0222643 31536591 PMC6752778

[57] Uddin J, Pulok M, Johnson R, Rana J, Baker E. Association between child marriage and institutional delivery care services use in Bangladesh: intersections between education and place of residence. Public Health. 2019;171:6–14. doi: 10.1016/j.puhe.2019.03.014 31071578

[58] Santhya K, Ram U, Acharya R, Jejeebhoy S, Ram F, Singh A. Associations Between Early Marriage and Young Women’s Marital and Reproductive Health Outcomes: Evidence from India. International perspectives on sexual and reproductive health. 2010;36:132–9. doi: 10.1363/ipsrh.36.132.10 20880798

[59] Trends in maternal mortality 2000 to 2020: estimates by WHO, UNICEF, UNFPA, World Bank Group and UNDESA/Population Division. Geneva: World Health Organization; 2023.

