# Supplementary material for: Associations between child marriage and reproductive and maternal health outcomes among young married women in Liberia and Sierra Leone: A cross-sectional study
Source: PLoS One. 2024 May 20;19(5):e0300982. doi: 10.1371/journal.pone.0300982 (PMC11104668; doi:10.1371/journal.pone.0300982)
Supplement: S1 Appendix — (DOCX) [file pone.0300982.s001.docx]

S1 Appendix. Adjusted odds ratios and 95% confidence intervals for full regression models of the association between child marriage and reproductive health outcomes, currently married women age 20-24, Liberia 2019-2020

|  | **Early Fertility** | |  | **High Fertility** | |  | **Low Fertility Control** | |
| --- | --- | --- | --- | --- | --- | --- | --- | --- |
| **Characteristics** | **AOR** | **95% CI** |  | **AOR** | **95% CI** |  | **AOR** | **95% CI** |
| **Age at first marriage** |  |  |  |  |  |  |  |  |
| Age 18 and older | 1.000 |  |  | 1.000 |  |  | 1.000 |  |
| Age 15-17 | 0.610 | [0.361,1.033] |  | 2.734** | [1.285,5.817] |  | 1.686 | [0.866,3.282] |
| Age <15 | 0.265** | [0.119,0.592] |  | 13.131*** | [5.855,29.450] |  | 3.935*** | [1.908,8.115] |
| **No. of decisions woman made alone or with husband** |  |  |  |  |  |  |  |  |
| **None** | 1.000 |  |  | 1.000 |  |  | 1.000 |  |
| 1 | 0.729 | [0.332,1.601] |  | 0.247 | [0.037,1.648] |  | 0.545 | [0.159,1.863] |
| 2 | 1.166 | [0.426,3.195] |  | 0.681 | [0.195,2.375] |  | 0.263* | [0.075,0.918] |
| 3 | 1.371 | [0.623,3.019] |  | 1.159 | [0.481,2.795] |  | 0.676 | [0.298,1.534] |
| **Woman has right to refuse sex if husband has an STI** |  |  |  |  |  |  |  |  |
| No | 1.000 |  |  | 1.000 |  |  | 1.000 |  |
| Yes | 1.177 | [0.614,2.256] |  | 0.743 | [0.315,1.754] |  | 1.525 | [0.776,2.995] |
| **Spouses’ relative education** |  |  |  |  |  |  |  |  |
| Same/woman higher | 1.000 |  |  | 1.000 |  |  | 1.000 |  |
| Husband higher | 0.755 | [0.442,1.289] |  | 0.588 | [0.270,1.284] |  | 0.643 | [0.304,1.359] |
| **Spouses’ relative age** |  |  |  |  |  |  |  |  |
| < 5 years | 1.000 |  |  | 1.000 |  |  | 1.000 |  |
| Husband 5-9 years older | 1.075 | [0.544,2.126] |  | 1.511 | [0.657,3.473] |  | 1.489 | [0.740,2.999] |
| Husband 10+ years older | 1.169 | [0.608,2.249] |  | 2.638** | [1.301,5.348] |  | 2.758* | [1.223,6.221] |
| **Woman’s age** | 1.134 | [0.973,1.322] |  | 2.125*** | [1.632,2.767] |  | 1.212 | [0.969,1.517] |
| **Woman’s education** |  |  |  |  |  |  |  |  |
| None | 1.000 |  |  | 1.000 |  |  | 1.000 |  |
| Primary | 0.948 | [0.503,1.786] |  | 2.048 | [0.949,4.419] |  | 1.756 | [0.713,4.326] |
| Secondary/higher | 0.733 | [0.342,1.573] |  | 0.809 | [0.353,1.857] |  | 1.332 | [0.595,2.979] |
| **Household wealth** |  |  |  |  |  |  |  |  |
| Low | 1.000 |  |  | 1.000 |  |  | 1.000 |  |
| Medium | 0.840 | [0.478,1.476] |  | 0.710 | [0.333,1.517] |  | 0.666 | [0.322,1.376] |
| High | 0.777 | [0.403,1.499] |  | 0.278** | [0.117,0.657] |  | 0.379** | [0.187,0.767] |
| **Religion** |  |  |  |  |  |  |  |  |
| Non-Muslim | 1.000 |  |  | 1.000 |  |  | 1.000 |  |
| Muslim | 0.753 | [0.361,1.573] |  | 1.483 | [0.625,3.517] |  | 1.318 | [0.562,3.094] |
| **Region** |  |  |  |  |  |  |  |  |
| Northwestern | 1.000 |  |  | 1.000 |  |  | 1.000 |  |
| South Central | 0.430 | [0.183,1.009] |  | 1.389 | [0.382,5.052] |  | 0.577 | [0.186,1.789] |
| Southeastern A | 0.337* | [0.147,0.771] |  | 0.770 | [0.171,3.471] |  | 0.511 | [0.167,1.569] |
| Southeastern B | 0.725 | [0.293,1.792] |  | 1.957 | [0.492,7.781] |  | 0.520 | [0.172,1.569] |
| North Central | 0.627 | [0.294,1.338] |  | 2.740 | [0.809,9.282] |  | 0.828 | [0.308,2.227] |
| **Type of Place of Residence** |  |  |  |  |  |  |  |  |
| Urban | 1.000 |  |  | 1.000 |  |  | 1.000 |  |
| Rural | 1.428 | [0.830,2.459] |  | 2.700** | [1.394,5.230] |  | 2.242* | [1.072,4.690] |
|  |  |  |  |  |  |  |  |  |
| **Number of Women** | **631** | |  | **631** | |  | **631** | |

* p<0.05, ** p<0.01, * ** p<0.001

S1 Appendix continued.

|  | **Unwanted/Mistimed Pregnancy** | |  | **Modern Contraceptive Use** | |
| --- | --- | --- | --- | --- | --- |
| **Characteristics** | **AOR** | **95% CI** |  | **AOR** | **95% CI** |
| **Age at first marriage** |  |  |  |  |  |
| Age 18 and older | 1.000 |  |  | 1.000 |  |
| Age 15-17 | 1.209 | [0.604,2.418] |  | 1.300 | [0.730,2.315] |
| Age <15 | 0.853 | [0.369,1.974] |  | 1.646 | [0.754,3.591] |
| **No. of decisions woman made alone or with husband** |  |  |  |  |  |
| **None** | 1.000 |  |  | 1.000 |  |
| 1 | 1.626 | [0.494,5.348] |  | 0.806 | [0.239,2.714] |
| 2 | 0.952 | [0.325,2.790] |  | 0.849 | [0.249,2.891] |
| 3 | 1.050 | [0.462,2.386] |  | 1.043 | [0.428,2.543] |
| **Woman has right to refuse sex if husband has an STI** |  |  |  |  |  |
| No | 1.000 |  |  | 1.000 |  |
| Yes | 1.001 | [0.554,1.809] |  | 1.294 | [0.645,2.597] |
| **Spouses’ relative education** |  |  |  |  |  |
| Same/woman higher | 1.000 |  |  | 1.000 |  |
| Husband higher | 0.860 | [0.487,1.520] |  | 1.005 | [0.596,1.695] |
| **Spouses’ relative age** |  |  |  |  |  |
| < 5 years | 1.000 |  |  | 1.000 |  |
| Husband 5-9 years older | 0.993 | [0.584,1.687] |  | 1.051 | [0.593,1.860] |
| Husband 10+ years older | 0.599 | [0.339,1.059] |  | 0.554 | [0.252,1.216] |
| **Woman’s age** | 0.903 | [0.779,1.048] |  | 0.957 | [0.798,1.148] |
| **Woman’s education** |  |  |  |  |  |
| None | 1.000 |  |  | 1.000 |  |
| Primary | 2.608** | [1.265,5.374] |  | 1.191 | [0.634,2.238] |
| Secondary/higher | 2.871* | [1.255,6.567] |  | 1.165 | [0.554,2.449] |
| **Household wealth** |  |  |  |  |  |
| Low | 1.000 |  |  | 1.000 |  |
| Medium | 1.236 | [0.728,2.100] |  | 2.816** | [1.419,5.591] |
| High | 0.826 | [0.460,1.484] |  | 1.334 | [0.631,2.821] |
| **Religion** |  |  |  |  |  |
| Non-Muslim | 1.000 |  |  | 1.000 |  |
| Muslim | 0.841 | [0.333,2.126] |  | 0.453 | [0.158,1.297] |
| **Region** |  |  |  |  |  |
| Northwestern | 1.000 |  |  | 1.000 |  |
| South Central | 1.703 | [0.698,4.155] |  | 0.463 | [0.169,1.267] |
| Southeastern A | 0.574 | [0.236,1.394] |  | 1.385 | [0.578,3.320] |
| Southeastern B | 0.625 | [0.218,1.793] |  | 1.941 | [0.811,4.647] |
| North Central | 0.885 | [0.404,1.937] |  | 0.353* | [0.143,0.871] |
| **Type of Place of Residence** |  |  |  |  |  |
| Urban | 1.000 |  |  | 1.000 |  |
| Rural | 1.156 | [0.677,1.975] |  | 0.519 | [0.252,1.070] |
| **No. of living sons** |  |  |  | 1.072 | [0.772,1.490] |
| **No. of FP message channels** |  |  |  | 1.207 | [0.753,1.935] |
|  |  |  |  |  |  |
| **Number of women** | **631** | |  | **631** | |

FP Family planning

* p<0.05; ** p<0.01; *** p<0.001
