# Supplementary material for: Associations between child marriage and reproductive and maternal health outcomes among young married women in Liberia and Sierra Leone: A cross-sectional study
Source: PLoS One. 2024 May 20;19(5):e0300982. doi: 10.1371/journal.pone.0300982 (PMC11104668; doi:10.1371/journal.pone.0300982)
Supplement: S2 Appendix — (DOCX) [file pone.0300982.s002.docx]

S2 Appendix. Adjusted odds ratios and 95% confidence intervals for full regression models of the association between child marriage and maternal health outcomes, currently married women age 20-24, Liberia 2019-2020

|  | **Four or More ANC Visits** | |  | **Skilled Attendant at Birth** | |  | **Institutional Delivery** | |
| --- | --- | --- | --- | --- | --- | --- | --- | --- |
| **Characteristics** | **AOR** | **95% CI** |  | **AOR** | **95% CI** |  | **AOR** | **95% CI** |
| **Age at first marriage** |  |  |  |  |  |  |  |  |
| Age 18 and older | 1.000 |  |  | 1.000 |  |  | 1.000 |  |
| Age 15-17 | 1.131 | [0.430,2.976] |  | 0.344*** | [0.184,0.643] |  | 0.582 | [0.259,1.308] |
| Age <15 | 0.520 | [0.151,4.276] |  | 0.361* | [0.142,0.921] |  | 0.702 | [0.284,1.736] |
| **No. of decisions woman made alone or with husband** |  |  |  |  |  |  |  |  |
| **None** | 1.000 |  |  | 1.000 |  |  | 1.000 |  |
| 1 | 0.803 | [0.151,4.277] |  | 1.644 | [0.519,5.206] |  | 0.636 | [0.153,2.648] |
| 2 | 0.942 | [0.245,3.625] |  | 0.495 | [0.168,1.458] |  | 0.427 | [0.108,1.686] |
| 3 | 0.990 | [0.303,3.242] |  | 1.170 | [0.421,3.251] |  | 0.759 | [0.217,2.653] |
| **Woman has right to refuse sex if husband has an STI** |  |  |  |  |  |  |  |  |
| No | 1.000 |  |  | 1.000 |  |  | 1.000 |  |
| Yes | 0.593 | [0.265,1.326] |  | 0.451 | [0.198,1.027] |  | 0.690 | [0.291,1.638] |
| **Spouses’ relative education** |  |  |  |  |  |  |  |  |
| Same/woman higher | 1.000 |  |  | 1.000 |  |  | 1.000 |  |
| Husband higher | 0.766 | [0.351,1.674] |  | 1.034 | [0.588,1.821] |  | 0.984 | [0.495,1.956] |
| **Spouses’ relative age** |  |  |  |  |  |  |  |  |
| < 5 years | 1.000 |  |  | 1.000 |  |  | 1.000 |  |
| Husband 5-9 years older | 0.515 | [0.182,1.462] |  | 0.951 | [0.469,1.930] |  | 0.562 | [0.261,1.208] |
| Husband 10+ years older | 0.574 | [0.191,1.720] |  | 1.390 | [0.629,3.073] |  | 1.113 | [0.428,2.898] |
| **Woman’s age** | 1.053 | [0.758,1.462] |  | 0.651*** | [0.516,0.821] |  | 0.745* | [0.565,0.982] |
| **Woman’s education** |  |  |  |  |  |  |  |  |
| None | 1.000 |  |  | 1.000 |  |  | 1.000 |  |
| Primary | 1.470 | [0.577,3.743] |  | 1.627 | [0.814,3.254] |  | 1.406 | [0.579,3.411] |
| Secondary/higher | 0.738 | [0.698,1.693] |  | 3.102* | [1.214,7.925] |  | 2.626 | [0.914,7.547] |
| **Household wealth** |  |  |  |  |  |  |  |  |
| Low | 1.000 |  |  | 1.000 |  |  | 1.000 |  |
| Medium | 1.241 | [0.782,1.970] |  | 0.741 | [0.387,1.420] |  | 0.727 | [0.309,1.708] |
| High | 1.189 | [0.721, 1.960] |  | 2.250 | [0.909,5.569] |  | 3.469* | [1.018,11.824 |
| **Religion** |  |  |  |  |  |  |  |  |
| Non-Muslim | 1.000 |  |  | 1.000 |  |  | 1.000 |  |
| Muslim | 1.081 | [0.653,1.788] |  | 1.379 | [0.472,4.031] |  | 0.676 | [0.219,2.087] |
| **Region** |  |  |  |  |  |  |  |  |
| Northwestern | 1.000 |  |  | 1.000 |  |  | 1.000 |  |
| South Central | 1.141 | [0.587,2.219] |  | 0.883 | [0.295,2.646] |  | 0.255* | [0.078,0.835] |
| Southeastern A | 0.657 | [0.378,1.142] |  | 1.734 | [0.554,5.427] |  | 0.689 | [0.158,2.999] |
| Southeastern B | 0.422** | [0.236,0.757] |  | 0.915 | [0.298,2.810] |  | 0.663 | [0.202,2.175] |
| North Central | 0.250*** | [0.118,0.531] |  | 2.120 | [0.844,5.328] |  | 2.218 | [0.647,7.608] |
| **Type of Place of Residence** |  |  |  |  |  |  |  |  |
| Urban | 1.000 |  |  | 1.000 |  |  | 1.000 |  |
| Rural | 0.590 | [0.251,1.385] |  | 0.595 | [0.286,1.234] |  | 0.361* | [0.163,0.801] |
| **Birth order** | 0.986 | [0.561,1.736] |  | 2.671*** | [1.806,3.951] |  | 1.441 | [0.941,2.207] |
|  |  |  |  |  |  |  |  |  |
| **Number of women** | **528** | |  | **528** | |  | **528** | |

* *p*<0.05, ** *p*<0.01, * ** *p*<0.001
