# Supplementary material for: Associations between child marriage and reproductive and maternal health outcomes among young married women in Liberia and Sierra Leone: A cross-sectional study
Source: PLoS One. 2024 May 20;19(5):e0300982. doi: 10.1371/journal.pone.0300982 (PMC11104668; doi:10.1371/journal.pone.0300982)
Supplement: S3 Appendix — (DOCX) [file pone.0300982.s003.docx]

S3 Appendix. Adjusted odds ratios and 95% confidence intervals for full regression models of the association between child marriage and reproductive health outcomes, currently married women age 20-24, Sierra Leone 2019

|  | **Early Fertility** | |  | **High Fertility** | |  | **Low Fertility Control** | |
| --- | --- | --- | --- | --- | --- | --- | --- | --- |
| **Characteristics** | **AOR** | **95% CI** |  | **AOR** | **95% CI** |  | **AOR** | **95% CI** |
| **Age at first marriage** |  |  |  |  |  |  |  |  |
| Age 18 and older | 1.000 |  |  | 1.000 |  |  | 1.000 |  |
| Age 15-17 | 0.485*** | [0.369,0.638] |  | 3.929*** | [2.397,6.439] |  | 1.728* | [1.139,2.622] |
| Age <15 | 0.347*** | [0.236,0.512] |  | 10.863*** | [6.436,18.336] |  | 3.447*** | [2.131,5.575] |
| **No. of decisions woman made alone or with husband/partner** |  |  |  |  |  |  |  |  |
| None | 1.000 |  |  | 1.000 |  |  | 1.000 |  |
| 1 | 0.918 | [0.628,1.343] |  | 0.313*** | [0.167,0.585] |  | 0.372** | [0.184,0.751] |
| 2 | 1.382 | [0.878,2.175] |  | 0.665 | [0.358,1.235] |  | 0.724 | [0.382,1.373] |
| 3 | 1.106 | [0.825,1.482] |  | 0.686 | [0.446,1.057] |  | 0.689 | [0.453,1.049] |
| **Woman has right to refuse sex if husband has an STI** |  |  |  |  |  |  |  |  |
| No | 1.000 |  |  | 1.000 |  |  | 1.000 |  |
| Yes | 0.988 | [0.729,1.338] |  | 0.867 | [0.599,1.255] |  | 1.077 | [0.727,1.594] |
| **Spouses’ relative education** |  |  |  |  |  |  |  |  |
| Same/woman higher | 1.000 |  |  | 1.000 |  |  | 1.000 |  |
| Husband higher | 1.254 | [0.919,1.710] |  | 1.217 | [0.822,1.801] |  | 1.295 | [0.907,1.849] |
| **Spouses’ relative age** |  |  |  |  |  |  |  |  |
| < 5 years | 1.000 |  |  | 1.000 |  |  | 1.000 |  |
| Husband 5-9 years older | 0.846 | [0.587,1.219] |  | 0.815 | [0.496,1.340] |  | 0.806 | [0.489,1.329] |
| Husband 10+ years older | 0.702* | [0.505,0.975] |  | 1.092 | [0.673,1.772] |  | 0.817 | [0.514,1.299] |
| **Woman’s age** | 0.976 | [0.890,1.071] |  | 1.784*** | [1.544,2.060] |  | 1.319*** | [1.161,1.500] |
| **Woman’s education** |  |  |  |  |  |  |  |  |
| None | 1.000 |  |  | 1.000 |  |  | 1.000 |  |
| Primary | 1.108 | [0.764,1.606] |  | 1.856* | [1.140,3.021] |  | 1.044 | [0.647,1.685] |
| Secondary/higher | 1.186 | [0.853,1.650] |  | 0.712 | [0.461,1.100] |  | 0.643 | [0.401,1.032] |
| **Household wealth** |  |  |  |  |  |  |  |  |
| Low | 1.000 |  |  | 1.000 |  |  | 1.000 |  |
| Medium | 0.975 | [0.717,1.325] |  | 0.705 | [0.444,1.122] |  | 0.858 | [0.573,1.283] |
| High | 0.930 | [0.654,1.322] |  | 0.506** | [0.311,0.824] |  | 0.741 | [0.464,1.184] |
| **Religion** |  |  |  |  |  |  |  |  |
| Non-Muslim | 1.000 |  |  | 1.000 |  |  | 1.000 |  |
| Muslim | 0.739 | [0.514,1.061] |  | 1.389 | [0.876,2.204] |  | 0.908 | [0.581,1.419] |
| **Region** |  |  |  |  |  |  |  |  |
| Eastern | 1.000 |  |  | 1.000 |  |  | 1.000 |  |
| Northern | 1.064 | [0.699,1.619] |  | 0.961 | [0.573,1.611] |  | 0.912 | [0.530,1.569] |
| Northwestern | 1.484 | [0.941,2.341] |  | 1.204 | [0.693,2.093] |  | 0.930 | [0.505,1.716] |
| Southern | 1.525* | [1.027,2.266] |  | 1.181 | [0.707,1.973] |  | 1.201 | [0.745,1.936] |
| Western | 1.189 | [0.693,2.038] |  | 1.251 | [0.557,2.808] |  | 0.735 | [0.333,1.624] |
| **Type of Place of Residence** |  |  |  |  |  |  |  |  |
| Urban | 1.000 |  |  | 1.000 |  |  | 1.000 |  |
| Rural | 1.279 | [0.818,1.998] |  | 2.717*** | [1.625,4.542] |  | 1.537 | [0.818,2.887] |
|  |  |  |  |  |  |  |  |  |
| **Number of women** | **1,325** | |  | **1,325** | |  | **1,325** | |

* *p*<0.05, ** *p*<0.01, * ** *p*<0.001

S3 Appendix Contd.

|  | **Unwanted/Mistimed Pregnancy** | |  | **Modern Contraceptive Use** | |
| --- | --- | --- | --- | --- | --- |
| **Characteristics** | **AOR** | **95% CI** |  | **AOR** | **95% CI** |
| **Age at first marriage** |  |  |  |  |  |
| Age 18 and older | 1.000 |  |  | 1.000 |  |
| Age 15-17 | 1.088 | [0.720,1.644] |  | 1.314 | [0.847,2.038] |
| Age <15 | 0.786 | [0.440,1.405] |  | 2.138** | [1.252,3.650] |
| **No. of decisions woman made alone or with husband/partner** |  |  |  |  |  |
| None | 1.000 |  |  | 1.000 |  |
| 1 | 1.634 | [0.950,2.810] |  | 1.461 | [0.823,2.594] |
| 2 | 1.906* | [1.017,3.572] |  | 1.522 | [0.851,2.723] |
| 3 | 0.651 | [0.398,1.064] |  | 1.332 | [0.898,1.976] |
| **Woman has right to refuse sex if husband has an STI** |  |  |  |  |  |
| No | 1.000 |  |  | 1.000 |  |
| Yes | 0.861 | [0.567,1.308] |  | 2.338*** | [1.473,3.712] |
| **Spouses’ relative education** |  |  |  |  |  |
| Same/woman higher | 1.000 |  |  | 1.000 |  |
| Husband higher | 1.266 | [0.836,1.918] |  | 1.423 | [0.992,2.042] |
| **Spouses’ relative age** |  |  |  |  |  |
| < 5 years | 1.000 |  |  | 1.000 |  |
| Husband 5-9 years older | 0.759 | [0.464,1.242] |  | 0.999 | [0.632,1.578] |
| Husband 10+ years older | 0.670 | [0.403,1.114] |  | 0.694 | [0.444,1.084] |
| **Woman’s age** | 1.032 | [0.893,1.192] |  | 1.109 | [0.989,1.244] |
| **Woman’s education** |  |  |  |  |  |
| None | 1.000 |  |  | 1.000 |  |
| Primary | 1.264 | [0.716,2.231] |  | 1.356 | [0.763,2.410] |
| Secondary/higher | 2.298** | [1.340,3.941] |  | 2.530*** | [1.599,4.003] |
| **Household wealth** |  |  |  |  |  |
| Low | 1.000 |  |  | 1.000 |  |
| Medium | 0.778 | [0.480,1.260] |  | 1.395 | [0.892,2.181] |
| High | 0.731 | [0.432,1.237] |  | 1.872* | [1.153,3.041] |
| **Religion** |  |  |  |  |  |
| Non-Muslim | 1.000 |  |  | 1.000 |  |
| Muslim | 0.902 | [0.588,1.382] |  | 0.731 | [0.462,1.157] |
| **Region** |  |  |  |  |  |
| Eastern | 1.000 |  |  | 1.000 |  |
| Northern | 1.067 | [0.566,2.011] |  | 0.899 | [0.533,1.517] |
| Northwestern | 1.570 | [0.828,2.977] |  | 0.625 | [0.354,1.103] |
| Southern | 1.361 | [0.748,2.477] |  | 1.299 | [0.789,2.138] |
| Western | 4.128*** | [2.103,8.105] |  | 0.663 | [0.337,1.304] |
| **Type of Place of Residence** |  |  |  |  |  |
| Urban | 1.000 |  |  | 1.000 |  |
| Rural | 2.339** | [1.352,4.046] |  | 0.535* | [0.327,0.877] |
| No. of living sons |  |  |  | 0.239 | [0.984,1.561] |
| No. of FP message channels |  |  |  | 0.069 | [0.790,1.448] |
|  |  |  |  |  |  |
| **Number of women** | **1,325** | |  | **1,325** | |

FP Family planning

* *p*<0.05, ** *p*<0.01, * ** *p*<0.001
