# Supplementary material for: Associations between child marriage and reproductive and maternal health outcomes among young married women in Liberia and Sierra Leone: A cross-sectional study
Source: PLoS One. 2024 May 20;19(5):e0300982. doi: 10.1371/journal.pone.0300982 (PMC11104668; doi:10.1371/journal.pone.0300982)
Supplement: S4 Appendix — (DOCX) [file pone.0300982.s004.docx]

S4 Appendix. Adjusted odds ratios and 95% confidence intervals for full regression models of the association between child marriage and maternal health outcomes, currently married women age 20-24, Sierra Leone 2019

|  | **Four or More ANC Visits** | |  | **Skilled Attendant at Birth** | |  | **Institutional Delivery** | |
| --- | --- | --- | --- | --- | --- | --- | --- | --- |
| **Characteristics** | **AOR** | **95% CI** |  | **AOR** | **95% CI** |  | **AOR** | **95% CI** |
| **Age at first marriage** |  |  |  |  |  |  |  |  |
| Age 18 and older | 1.000 |  |  | 1.000 |  |  | 1.000 |  |
| Age 15-17 | 1.090 | [0.756,1.572] |  | 1.170 | [0.670,2.040] |  | 1.051 | [0.653,1.693] |
| Age <15 | 0.926 | [0.541,1.585] |  | 1.213 | [0.612,2.401] |  | 1.043 | [0.567,1.919] |
| **No. of decisions woman made alone or with husband/partner** |  |  |  |  |  |  |  |  |
| None | 1.000 |  |  | 1.000 |  |  | 1.000 |  |
| 1 | 0.761 | [0.453,1.278] |  | 0.773 | [0.411,1.452] |  | 0.573* | [0.330,0.996] |
| 2 | 0.818 | [0.467,1.431] |  | 1.774 | [0.725,4.343] |  | 1.188 | [0.556,2.536] |
| 3 | 1.015 | [0.634,1.624] |  | 1.170 | [0.682, 2.007] |  | 1.053 | [0.645,1.719] |
| **Woman has right to refuse sex if husband has an STI** |  |  |  |  |  |  |  |  |
| No | 1.000 |  |  | 1.000 |  |  | 1.000 |  |
| Yes | 1.393 | [0.956,2.031] |  | 0.607* | [0.383, .960] |  | 0.538** | [0.353,0.820] |
| **Spouses’ relative education** |  |  |  |  |  |  |  |  |
| Same/woman higher | 1.000 |  |  | 1.000 |  |  | 1.000 |  |
| Husband higher | 0.938 | [0.632,1.393] |  | 1.318 | [0.722,2.407] |  | 1.087 | [0.651,1.815] |
| **Spouses’ relative age** |  |  |  |  |  |  |  |  |
| < 5 years | 1.000 |  |  | 1.000 |  |  | 1.000 |  |
| Husband 5-9 years older | 0.763 | [0.447,1.303 |  | 1.071 | [0.583,1.968] |  | 0.993 | [0.581,1.695] |
| Husband 10+ years older | 0.716 | [0.453,1.130] |  | 1.106 | [0.604,2.022] |  | 1.051 | [0.604,1.829] |
| **Woman’s age** | 0.945 | [0.828,1.077] |  | 0.967 | [0.799,1.171] |  | 0.934 | [0.797,1.093] |
| **Woman’s education** |  |  |  |  |  |  |  |  |
| None | 1.000 |  |  | 1.000 |  |  | 1.000 |  |
| Primary | 0.785 | [0.476,1.295] |  | 1.469 | 0.871, 2.478] |  | 1.676 | [0.992,2.830] |
| Secondary/higher | 1.087 | [0.698,1.693] |  | 2.906*** | [1.591, 5.308] |  | 2.817*** | [1.668,4.757] |
| **Household wealth** |  |  |  |  |  |  |  |  |
| Low | 1.000 |  |  | 1.000 |  |  | 1.000 |  |
| Medium | 1.241 | [0.782,1.970] |  | 0.916 | [0.535,1.568] |  | 0.948 | [0.584,1.540] |
| High | 1.189 | [0.721, 1.960] |  | 0.867 | [0.444,1.693] |  | 1.265 | [0.673,2.378] |
| **Religion** |  |  |  |  |  |  |  |  |
| Non-Muslim | 1.000 |  |  | 1.000 |  |  | 1.000 |  |
| Muslim | 1.081 | [0.653,1.788] |  | 1.063 | [0.548, 2.061] |  | 1.04 | [0.542,1.995] |
| **Region** |  |  |  |  |  |  |  |  |
| Eastern | 1.000 |  |  | 1.000 |  |  | 1.000 |  |
| Northern | 1.141 | [0.587,2.219] |  | 0.396* | [0.175,0.895] |  | 0.295*** | [0.144,0.606] |
| Northwestern | 0.657 | [0.378,1.142] |  | 0.195*** | [0.086,0.439] |  | 0.207*** | [0.099,0.433] |
| Southern | 0.422** | [0.236,0.757] |  | 0.636 | [0.256,1.584] |  | 0.798 | [0.324,1.969] |
| Western | 0.250*** | [0.118,0.531] |  | 0.360 | [0.118,1.100] |  | 0.269** | [0.110,0.661] |
| **Type of Place of Residence** |  |  |  |  |  |  |  |  |
| Urban | 1.000 |  |  | 1.000 |  |  | 1.000 |  |
| Rural | 1.042 | [0.846,1.347] |  | 0.373* | [0.161,0.862] |  | 0.755 | [0.418,1.363] |
| Birth order | 1.068 | [0.860,1.347] |  | 0.872 | [0.626,1.213] |  | 0.917 | [0.691,1.218] |
|  |  |  |  |  |  |  |  |  |
| **Number of women** | **1,093** | |  | **1,093** | |  | **1,093** | |

FP Family planning

* *p*<0.05, ** *p*<0.01, * ** *p*<0.001
