# Supplementary material for: Associations between child marriage and reproductive and maternal health outcomes among young married women in Liberia and Sierra Leone: A cross-sectional study
Source: PLoS One. 2024 May 20;19(5):e0300982. doi: 10.1371/journal.pone.0300982 (PMC11104668; doi:10.1371/journal.pone.0300982)
Supplement: S5 Appendix — (DOCX) [file pone.0300982.s005.docx]

S5 Appendix. Adjusted odds ratios and 95% confidence intervals for the association between child marriage and reproductive health outcomes after adjusting for other factors, currently married women aged 20-24, Sierra Leone 2019 and Liberia 2019-2020 Combined

|  | **Early Fertility** | |  | **High Fertility** | |  | **Low Fertility Control** | |  | **Has Had a Terminated Pregnancy** | |
| --- | --- | --- | --- | --- | --- | --- | --- | --- | --- | --- | --- |
| **Characteristics** | **AOR** | **95% CI** |  | **AOR** | **95% CI** |  | **AOR** | **95% CI** |  |  |  |
| **Age at first marriage** |  |  |  |  |  |  |  |  |  |  |  |
| Age 18 and older | 1.000 |  |  | 1.000 |  |  | 1.000 |  |  | 1.000 |  |
| Age 15-17 | 0.543*** | [0.428,0.691] |  | 3.620*** | [2.405,5.448] |  | 1.696** | [1.202,2.393] |  | 0.804 | [0.521,1.240] |
| Age <15 | 0.325*** | [0.233,0.455] |  | 10.673*** | [7.128,15.980] |  | 3.198*** | [2.178,4.695] |  | 0.918 | [0.576,1.464] |
| **Country** |  |  |  |  |  |  |  |  |  |  |  |
| Liberia | 1.000 |  |  | 1.000 |  |  | 1.000 |  |  | 1.000 |  |
| Sierra Leone | 0.914 | [0.660,1.266] |  | 0.736 | [0.478,1.133] |  | 0.696 | [0.429,1.129] |  | 0.576* | [0.349,0.951] |
| **No. of decisions woman made alone or with husband/partner** |  |  |  |  |  |  |  |  |  |  |  |
| None | 1.000 |  |  | 1.000 |  |  | 1.000 |  |  | 1.000 |  |
| 1 | 0.875 | [0.631,1.214] |  | 0.324*** | [0.187,0.561] |  | 0.414** | [0.235,0.728] |  | 1.277 | [0.716,2.277] |
| 2 | 1.364 | [0.937,1.984] |  | 0.597* | [0.357,1.000] |  | 0.517* | [0.296,0.902] |  | 1.273 | [0.693,2.339] |
| 3 | 1.198 | [0.916,1.568] |  | 0.769 | [0.537,1.100] |  | 0.664* | [0.462,0.954] |  | 1.336 | [0.837,2.132] |
| **Woman has right to refuse sex if husband has an STI** |  |  |  |  |  |  |  |  |  |  |  |
| No | 1.000 |  |  | 1.000 |  |  | 1.000 |  |  | 1.000 |  |
| Yes | 1.024 | [0.782,1.341] |  | 0.846 | [0.592,1.208] |  | 1.219 | [0.880,1.690] |  | 1.350 | [0.769,2.369] |
| **Spouses’ relative education** |  |  |  |  |  |  |  |  |  |  |  |
| Same/woman higher | 1.000 |  |  | 1.000 |  |  | 1.000 |  |  | 1.000 |  |
| Husband higher | 1.09 | [0.844,1.407] |  | 0.964 | [0.673,1.381] |  | 1.065 | [0.754,1.506] |  | 1.168 | [0.766,1.780] |
| **Spouses’ relative age** |  |  |  |  |  |  |  |  |  |  |  |
| < 5 years | 1.000 |  |  | 1.000 |  |  | 1.000 |  |  | 1.000 |  |
| Husband 5-9 years older | 0.936 | [0.677,1.295] |  | 1.031 | [0.672,1.581] |  | 1.067 | [0.709,1.607] |  | 0.967 | [0.600,1.559] |
| Husband 10+ years older | 0.818 | [0.615,1.086] |  | 1.375 | [0.941,2.011] |  | 1.140 | [0.746,1.742] |  | 1.145 | [0.707,1.854] |
| **Woman’s age** | 1.004 | [0.931,1.083] |  | 1.865*** | [1.649,2.108] |  | 1.288*** | [1.155,1.436] |  | 1.065 | [0.924,1.227] |
| **Woman’s education** |  |  |  |  |  |  |  |  |  |  |  |
| None | 1.000 |  |  | 1.000 |  |  | 1.000 |  |  | 1.000 |  |
| Primary | 1.057 | [0.774,1.442] |  | 1.695** | [1.145,2.508] |  | 1.104 | [0.738,1.651] |  | 1.108 | [0.634,1.936] |
| Secondary/higher | 1.043 | [0.775,1.404] |  | 0.704 | [0.486,1.021] |  | 0.754 | [0.505,1.128] |  | 0.596 | [0.346,1.028] |
| **Household wealth** |  |  |  |  |  |  |  |  |  |  |  |
| Low | 1.000 |  |  | 1.000 |  |  | 1.000 |  |  | 1.000 |  |
| Medium | 0.893 | [0.688,1.159] |  | 0.713 | [0.489,1.041] |  | 0.752 | [0.528,1.071] |  | 0.827 | [0.531,1.286] |
| High | 0.894 | [0.669,1.195] |  | 0.434*** | [0.286,0.659] |  | 0.614* | [0.420,0.900] |  | 0.931 | [0.595,1.457] |
| **Religion** |  |  |  |  |  |  |  |  |  |  |  |
| Non-Muslim | 1.000 |  |  | 1.000 |  |  | 1.000 |  |  | 1.000 |  |
| Muslim | 0.827 | [0.613,1.115] |  | 1.296 | [0.880,1.910] |  | 1.032 | [0.691,1.542] |  | 1.078 | [0.678,1.715] |
| **Type of place of residence** |  |  |  |  |  |  |  |  |  |  |  |
| Urban | 1.000 |  |  | 1.000 |  |  | 1.000 |  |  | 1.000 |  |
| Rural | 1.330* | [1.007,1.758] |  | 2.737*** | [1.938,3.865] |  | 2.020*** | [1.350,3.021] |  | 0.593* | [0.383,0.919] |
|  |  |  |  |  |  |  |  |  |  |  |  |
| **Number of Cases** | **1997** |  |  | **1997** |  |  | **1997** |  |  | **1997** |  |

Note: All regression models control for region.

* *p*<0.05, ** *p*<0.01, * ** *p*<0.001

|  | **Unwanted Pregnancy** | |  | **Multiple Unwanted Pregnancy** | |  | **Modern Contraceptive Use** | |
| --- | --- | --- | --- | --- | --- | --- | --- | --- |
| **Characteristics** | **AOR** | **95% CI** |  | **AOR** | **95% CI** |  | **AOR** | **95% CI** |
| **Age at first marriage** |  |  |  |  |  |  |  |  |
| Age 18 and older | 1.000 |  |  | 1.000 |  |  | 1.000 |  |
| Age 15-17 | 1.104 | [0.767,1.589] |  | 2.597 | [0.882,7.642] |  | 1.336 | [0.959,1.862] |
| Age <15 | 0.732 | [0.472,1.134] |  | 1.219 | [0.365,4.065] |  | 2.050*** | [1.367,3.074] |
| **Country** |  |  |  |  |  |  |  |  |
| Liberia | 1.000 |  |  | 1.000 |  |  | 1.000 |  |
| Sierra Leone | 0.356*** | [0.242,0.525] |  | 0.139*** | [0.058,0.333] |  | 0.816 | [0.538,1.239] |
| **No. of decisions woman made alone or with husband** |  |  |  |  |  |  |  |  |
| **None** | 1.000 |  |  | 1.000 |  |  | 1.000 |  |
| 1 | 1.565 | [0.974,2.515] |  | 4.918* | [1.234,19.606] |  | 1.274 | [0.784,2.070] |
| 2 | 1.563 | [0.932,2.621] |  | 1.952 | [0.697,5.468] |  | 1.343 | [0.830,2.175] |
| 3 | 0.899 | [0.622,1.299] |  | 1.159 | [0.435,3.089] |  | 1.342 | [0.946,1.903] |
| **Woman has right to refuse sex if husband has an STI** |  |  |  |  |  |  |  |  |
| No | 1.000 |  |  | 1.000 |  |  | 1.000 |  |
| Yes | 0.922 | [0.661,1.287] |  | 0.801 | [0.303,2.114] |  | 1.852*** | [1.297,2.645] |
| **Spouses’ relative education** |  |  |  |  |  |  |  |  |
| Same/woman higher | 1.000 |  |  | 1.000 |  |  | 1.000 |  |
| Husband higher | 1.028 | [0.742,1.424] |  | 0.707 | [0.347,1.440] |  | 1.279 | [0.956,1.711] |
| **Spouses’ relative age** |  |  |  |  |  |  |  |  |
| < 5 years | 1.000 |  |  | 1.000 |  |  | 1.000 |  |
| Husband 5-9 years older | 0.887 | [0.631,1.245] |  | 2.100* | [1.121,3.935] |  | 0.988 | [0.697,1.401] |
| Husband 10+ years older | 0.618** | [0.430,0.887] |  | 0.914 | [0.379,2.205] |  | 0.674* | [0.461,0.985] |
| **Woman’s age** | 0.991 | [0.899,1.092] |  | 1.218 | [0.972,1.526] |  | 1.025 | [0.934,1.126] |
| **Woman’s education** |  |  |  |  |  |  |  |  |
| None | 1.000 |  |  | 1.000 |  |  | 1.000 |  |
| Primary | 1.566* | [1.025,2.394] |  | 3.482* | [1.076,11.271] |  | 1.357 | [0.902,2.044 |
| Secondary/higher | 2.241*** | [1.470,3.417] |  | 3.931* | [1.238,12.479] |  | 1.955*** | [1.326,2.881 |
| **Household wealth** |  |  |  |  |  |  |  |  |
| Low | 1.000 |  |  | 1.000 |  |  | 1.000 |  |
| Medium | 1.055 | [0.747,1.491] |  | 0.952 | [0.502,1.805] |  | 1.709** | [1.205,2.423] |
| High | 0.959 | [0.656,1.402] |  | 0.557 | [0.237,1.309] |  | 1.620* | [1.103,2.380] |
| **Religion** |  |  |  |  |  |  |  |  |
| Non-Muslim | 1.000 |  |  | 1.000 |  |  | 1.000 |  |
| Muslim | 0.883 | [0.608,1.284] |  | 0.653 | [0.295,1.442] |  | 0.614* | [0.421,0.895] |
| **Type of Place of Residence** |  |  |  |  |  |  |  |  |
| Urban | 1.000 |  |  | 1.000 |  |  | 1.000 |  |
| Rural | 1.105 | [0.808,1.510] |  | 1.166 | [0.497,2.733] |  | 0.529*** | [0.367,0.762] |
| **No. of living sons** |  |  |  |  |  |  | 1.183 | [0.987,1.418] |
| **No. of FP message channels** |  |  |  |  |  |  | 1.167 | [0.926,1.470] |
|  |  |  |  |  |  |  |  |  |
| **Number of Cases** | **1997** |  |  | **1997** |  |  | **1997** |  |

Note: All regression models control for region.

* *p*<0.05, ** *p*<0.01, * ** *p*<0.001
