# Supplementary material for: Associations between child marriage and reproductive and maternal health outcomes among young married women in Liberia and Sierra Leone: A cross-sectional study
Source: PLoS One. 2024 May 20;19(5):e0300982. doi: 10.1371/journal.pone.0300982 (PMC11104668; doi:10.1371/journal.pone.0300982)
Supplement: S6 Appendix — (DOCX) [file pone.0300982.s006.docx]

S6 Appendix. Adjusted odds ratios and 95% confidence intervals for the association between child marriage and maternal health outcomes after adjusting for other factors, currently married women aged 20-24, Sierra Leone 2019 and Liberia 2019-2020 Combined

|  | **Four or More ANC Visits** | |  | **Skilled Attendant at Birth** | |  | **Institutional Delivery** | |
| --- | --- | --- | --- | --- | --- | --- | --- | --- |
| **Characteristics** | **AOR** | **95% CI** |  | **AOR** | **95% CI** |  | **AOR** | **95% CI** |
| **Age at first marriage** |  |  |  |  |  |  |  |  |
| Age 18 and older | 1.000 |  |  | 1.000 |  |  | 1.000 |  |
| Age 15-17 | 0.838 | [0.537,1.310] |  | 0.575** | [0.403,0.820] |  | 0.866 | [0.578,1.299] |
| Age <15 | 0.553 | [0.304,1.008] |  | 0.361*** | [0.222,0.587] |  | 0.944 | [0.574,1.552] |
| **Country** |  |  |  |  |  |  |  |  |
| Liberia | 1.000 |  |  | 1.000 |  |  | 1.000 |  |
| Sierra Leone | 0.976 | [0.521,1.827] |  | 1.022 | [0.674,1.550] |  | 1.113 | [0.649,1.908] |
| **No. of decisions woman made alone or with husband/partner** |  |  |  |  |  |  |  |  |
| None | 1.000 |  |  | 1.000 |  |  | 1.000 |  |
| 1 | 1.038 | [0.560,1.926] |  | 1.011 | [0.652,1.569] |  | 0.622 | [0.374,1.033] |
| 2 | 1.563 | [0.852,2.868] |  | 1.093 | [0.642,1.862] |  | 0.911 | [0.492,1.687] |
| 3 | 1.671 | [0.986,2.832] |  | 1.107 | [0.770,1.589] |  | 1.021 | [0.676,1.542] |
| **Woman has right to refuse sex if husband has an STI** |  |  |  |  |  |  |  |  |
| No | 1.000 |  |  | 1.000 |  |  | 1.000 |  |
| Yes | 1.041 | [0.676,1.603] |  | 0.480*** | [0.334,0.689] |  | 0.544** | [0.376,0.786] |
| **Spouses’ relative education** |  |  |  |  |  |  |  |  |
| Same/woman higher | 1.000 |  |  | 1.000 |  |  | 1.000 |  |
| Husband higher | 1.058 | [0.662,1.692] |  | 1.023 | [0.737,1.421] |  | 1.020 | [0.688,1.514] |
| **Spouses’ relative age** |  |  |  |  |  |  |  |  |
| < 5 years | 1.000 |  |  | 1.000 |  |  | 1.000 |  |
| Husband 5-9 years older | 0.779 | [0.449,1.353] |  | 1.214 | [0.797,1.848] |  | 0.827 | [0.531,1.288] |
| Husband 10+ years older | 0.779 | [0.471,1.288] |  | 1.248 | [0.810,1.922] |  | 1.097 | [0.688,1.750] |
| **Woman’s age** | 1.034 | [0.875,1.222] |  | 0.767*** | [0.676,0.869] |  | 0.851* | [0.744,0.974] |
| **Woman’s education** |  |  |  |  |  |  |  |  |
| None | 1.000 |  |  | 1.000 |  |  | 1.000 |  |
| Primary | 0.797 | [0.464,1.369] |  | 1.145 | [0.801,1.635] |  | 1.597* | [1.074,2.374] |
| Secondary/higher | 0.955 | [0.586,1.556] |  | 2.513*** | [1.696,3.725] |  | 2.827*** | [1.809,4.418] |
| **Household wealth** |  |  |  |  |  |  |  |  |
| Low | 1.000 |  |  | 1.000 |  |  | 1.000 |  |
| Medium | 1.279 | [0.763,2.141] |  | 0.758 | [0.530,1.084] |  | 0.795 | [0.531,1.191] |
| High | 1.418 | [0.784,2.566] |  | 0.937 | [0.636,1.381] |  | 1.294 | [0.804,2.083] |
| **Religion** |  |  |  |  |  |  |  |  |
| Non-Muslim | 1.000 |  |  | 1.000 |  |  | 1.000 |  |
| Muslim | 1.056 | [0.613,1.818] |  | 1.280 | [0.863,1.898] |  | 1.007 | [0.588,1.727] |
| **Type of place of residence** |  |  |  |  |  |  |  |  |
| Urban | 1.000 |  |  | 1.000 |  |  | 1.000 |  |
| Rural | 0.981 | [0.594,1.621] |  | 0.757 | [0.514,1.117] |  | 0.803 | [0.524,1.230] |
| **Birth order** | 0.972 | [0.770,1.228] |  | 1.889*** | [1.438,2.482] |  | 1.053 | [0.839,1.323] |
|  |  |  |  |  |  |  |  |  |
| **Number of Cases** | **1521** |  |  | **1789** |  |  | **1655** |  |

Note: All regression models control for region.

FP Family planning

* *p*<0.05, ** *p*<0.01, * ** *p*<0.001
