# Supplementary material for: Associations between child marriage and reproductive and maternal health outcomes among young married women in Liberia and Sierra Leone: A cross-sectional study
Source: PLoS One. 2024 May 20;19(5):e0300982. doi: 10.1371/journal.pone.0300982 (PMC11104668; doi:10.1371/journal.pone.0300982)
Supplement: S7 Appendix — (DOCX) [file pone.0300982.s007.docx]

S7 Appendix. Adjusted odds ratios and confidence intervals from regressions of the association of child marriage with reproductive and maternal health outcomes, currently married women aged 20-24, Sierra Leone 2019 and Liberia 2019-2020

|  |  | **Liberia** | |  | **Sierra Leone** | |
| --- | --- | --- | --- | --- | --- | --- |
| **Outcome** |  | **15-17** | **<15** |  | **15-17** | **<15** |
| Early fertility |  | 0.61 [0.36,1.03] | 0.27** [0.12,0.59] |  | 0.49*** 0.37,0.64] | 0.347*** [0.24,0.5] |
| High fertility |  | 2.73** [1.29,5.82] | 13.13*** [5.86,29.45] |  | 3.93*** [2.40,6.44] | 10.86***[6.44,18.34] |
| Low fertility control |  | 1.69 [0.87,3.28] | 3.94*** [1.91,8.12] |  | 1.73* [1.14,2.62] | 3.45*** [2.13,5.58] |
| Ever had terminated pregnancy |  | 0.92 [0.43,1.97] | 0.74 [0.31,1.79] |  | 0.67 [0.39,1.14] | 0.98 [0.54,1.78] |
| Unwanted pregnancy |  | 1.21 [0.60,2.42] | 0.85 [0.37,1.97] |  | 1.09 [0.72,1.64] | 0.79 [0.44,1.41] |
| Multiple unwanted pregnancies |  | 2.258 [0.58,8.84] | 0.78 [0.14,4.42] |  | 3.80 [0.74,19.4] | 3.86 [0.78,19.15] |
| Modern contraceptive use |  | 1.30 [0.73,2.32] | 1.65 [0.75,3.59] |  | 1.31 [0.85,2.04] | 2.14** [1.25,3.65] |
| Four or more ANC visits ^a^ |  | 0.72 [0.21,2.47] | 0.31 [0.08,1.19] |  | 0.89 [0.545,1.466] | 0.697 [0.344,1.412] |
| Skilled personnel at delivery ^a^ |  | 0.34*** [0.18,0.64] | 0.36* [0.14,0.92] |  | 0.74 [0.48,1.14] | 0.37*** [0.21,0.66] |
| Institutional delivery ^a^ |  | 0.58 [0.26,1.31] | 0.70 [0.28,1.74] |  | 1.05 [0.65,1.69] | 1.04 [0.57,1.92] |

Notes: Data were weighted. All models controlled for age, type of place of residence, household wealth, woman’s level of education, partner age gap, spousal education gap, religion, ability to negotiate safe sex, and region of residence. For modern contraceptive use, regressions also control for number of living sons, and number of family planning message channels. The age at marriage reference categories is 18 years or older.

^a^ Restricted to women with a live birth in the past five years

* Significant at *p* < .05; ** *p* < .005
