# Supplementary material for: Associations between child marriage and reproductive and maternal health outcomes among young married women in Liberia and Sierra Leone: A cross-sectional study
Source: PLoS One. 2024 May 20;19(5):e0300982. doi: 10.1371/journal.pone.0300982 (PMC11104668; doi:10.1371/journal.pone.0300982)
Supplement: S1 File — (DOCX) [file pone.0300982.s008.docx]

S1 Appendix. Adjusted odds ratios and 95% confidence intervals for full regression models of the association between child marriage and reproductive health outcomes, currently married women age 20-24, Liberia 2019-2020

|  | **Early Fertility** | |  | **High Fertility** | |  | **Low Fertility Control** | |
| --- | --- | --- | --- | --- | --- | --- | --- | --- |
| **Characteristics** | **AOR** | **95% CI** |  | **AOR** | **95% CI** |  | **AOR** | **95% CI** |
| **Age at first marriage** |  |  |  |  |  |  |  |  |
| Age 18 and older | 1.000 |  |  | 1.000 |  |  | 1.000 |  |
| Age 15-17 | 0.610 | [0.361,1.033] |  | 2.734** | [1.285,5.817] |  | 1.686 | [0.866,3.282] |
| Age <15 | 0.265** | [0.119,0.592] |  | 13.131*** | [5.855,29.450] |  | 3.935*** | [1.908,8.115] |
| **No. of decisions woman made alone or with husband** |  |  |  |  |  |  |  |  |
| **None** | 1.000 |  |  | 1.000 |  |  | 1.000 |  |
| 1 | 0.729 | [0.332,1.601] |  | 0.247 | [0.037,1.648] |  | 0.545 | [0.159,1.863] |
| 2 | 1.166 | [0.426,3.195] |  | 0.681 | [0.195,2.375] |  | 0.263* | [0.075,0.918] |
| 3 | 1.371 | [0.623,3.019] |  | 1.159 | [0.481,2.795] |  | 0.676 | [0.298,1.534] |
| **Woman has right to refuse sex if husband has an STI** |  |  |  |  |  |  |  |  |
| No | 1.000 |  |  | 1.000 |  |  | 1.000 |  |
| Yes | 1.177 | [0.614,2.256] |  | 0.743 | [0.315,1.754] |  | 1.525 | [0.776,2.995] |
| **Spouses’ relative education** |  |  |  |  |  |  |  |  |
| Same/woman higher | 1.000 |  |  | 1.000 |  |  | 1.000 |  |
| Husband higher | 0.755 | [0.442,1.289] |  | 0.588 | [0.270,1.284] |  | 0.643 | [0.304,1.359] |
| **Spouses’ relative age** |  |  |  |  |  |  |  |  |
| < 5 years | 1.000 |  |  | 1.000 |  |  | 1.000 |  |
| Husband 5-9 years older | 1.075 | [0.544,2.126] |  | 1.511 | [0.657,3.473] |  | 1.489 | [0.740,2.999] |
| Husband 10+ years older | 1.169 | [0.608,2.249] |  | 2.638** | [1.301,5.348] |  | 2.758* | [1.223,6.221] |
| **Woman’s age** | 1.134 | [0.973,1.322] |  | 2.125*** | [1.632,2.767] |  | 1.212 | [0.969,1.517] |
| **Woman’s education** |  |  |  |  |  |  |  |  |
| None | 1.000 |  |  | 1.000 |  |  | 1.000 |  |
| Primary | 0.948 | [0.503,1.786] |  | 2.048 | [0.949,4.419] |  | 1.756 | [0.713,4.326] |
| Secondary/higher | 0.733 | [0.342,1.573] |  | 0.809 | [0.353,1.857] |  | 1.332 | [0.595,2.979] |
| **Household wealth** |  |  |  |  |  |  |  |  |
| Low | 1.000 |  |  | 1.000 |  |  | 1.000 |  |
| Medium | 0.840 | [0.478,1.476] |  | 0.710 | [0.333,1.517] |  | 0.666 | [0.322,1.376] |
| High | 0.777 | [0.403,1.499] |  | 0.278** | [0.117,0.657] |  | 0.379** | [0.187,0.767] |
| **Religion** |  |  |  |  |  |  |  |  |
| Non-Muslim | 1.000 |  |  | 1.000 |  |  | 1.000 |  |
| Muslim | 0.753 | [0.361,1.573] |  | 1.483 | [0.625,3.517] |  | 1.318 | [0.562,3.094] |
| **Region** |  |  |  |  |  |  |  |  |
| Northwestern | 1.000 |  |  | 1.000 |  |  | 1.000 |  |
| South Central | 0.430 | [0.183,1.009] |  | 1.389 | [0.382,5.052] |  | 0.577 | [0.186,1.789] |
| Southeastern A | 0.337* | [0.147,0.771] |  | 0.770 | [0.171,3.471] |  | 0.511 | [0.167,1.569] |
| Southeastern B | 0.725 | [0.293,1.792] |  | 1.957 | [0.492,7.781] |  | 0.520 | [0.172,1.569] |
| North Central | 0.627 | [0.294,1.338] |  | 2.740 | [0.809,9.282] |  | 0.828 | [0.308,2.227] |
| **Type of Place of Residence** |  |  |  |  |  |  |  |  |
| Urban | 1.000 |  |  | 1.000 |  |  | 1.000 |  |
| Rural | 1.428 | [0.830,2.459] |  | 2.700** | [1.394,5.230] |  | 2.242* | [1.072,4.690] |
|  |  |  |  |  |  |  |  |  |
| **Number of Women** | **631** | |  | **631** | |  | **631** | |

* p<0.05, ** p<0.01, * ** p<0.001

S1 Appendix continued.

|  | **Unwanted/Mistimed Pregnancy** | |  | **Modern Contraceptive Use** | |
| --- | --- | --- | --- | --- | --- |
| **Characteristics** | **AOR** | **95% CI** |  | **AOR** | **95% CI** |
| **Age at first marriage** |  |  |  |  |  |
| Age 18 and older | 1.000 |  |  | 1.000 |  |
| Age 15-17 | 1.209 | [0.604,2.418] |  | 1.300 | [0.730,2.315] |
| Age <15 | 0.853 | [0.369,1.974] |  | 1.646 | [0.754,3.591] |
| **No. of decisions woman made alone or with husband** |  |  |  |  |  |
| **None** | 1.000 |  |  | 1.000 |  |
| 1 | 1.626 | [0.494,5.348] |  | 0.806 | [0.239,2.714] |
| 2 | 0.952 | [0.325,2.790] |  | 0.849 | [0.249,2.891] |
| 3 | 1.050 | [0.462,2.386] |  | 1.043 | [0.428,2.543] |
| **Woman has right to refuse sex if husband has an STI** |  |  |  |  |  |
| No | 1.000 |  |  | 1.000 |  |
| Yes | 1.001 | [0.554,1.809] |  | 1.294 | [0.645,2.597] |
| **Spouses’ relative education** |  |  |  |  |  |
| Same/woman higher | 1.000 |  |  | 1.000 |  |
| Husband higher | 0.860 | [0.487,1.520] |  | 1.005 | [0.596,1.695] |
| **Spouses’ relative age** |  |  |  |  |  |
| < 5 years | 1.000 |  |  | 1.000 |  |
| Husband 5-9 years older | 0.993 | [0.584,1.687] |  | 1.051 | [0.593,1.860] |
| Husband 10+ years older | 0.599 | [0.339,1.059] |  | 0.554 | [0.252,1.216] |
| **Woman’s age** | 0.903 | [0.779,1.048] |  | 0.957 | [0.798,1.148] |
| **Woman’s education** |  |  |  |  |  |
| None | 1.000 |  |  | 1.000 |  |
| Primary | 2.608** | [1.265,5.374] |  | 1.191 | [0.634,2.238] |
| Secondary/higher | 2.871* | [1.255,6.567] |  | 1.165 | [0.554,2.449] |
| **Household wealth** |  |  |  |  |  |
| Low | 1.000 |  |  | 1.000 |  |
| Medium | 1.236 | [0.728,2.100] |  | 2.816** | [1.419,5.591] |
| High | 0.826 | [0.460,1.484] |  | 1.334 | [0.631,2.821] |
| **Religion** |  |  |  |  |  |
| Non-Muslim | 1.000 |  |  | 1.000 |  |
| Muslim | 0.841 | [0.333,2.126] |  | 0.453 | [0.158,1.297] |
| **Region** |  |  |  |  |  |
| Northwestern | 1.000 |  |  | 1.000 |  |
| South Central | 1.703 | [0.698,4.155] |  | 0.463 | [0.169,1.267] |
| Southeastern A | 0.574 | [0.236,1.394] |  | 1.385 | [0.578,3.320] |
| Southeastern B | 0.625 | [0.218,1.793] |  | 1.941 | [0.811,4.647] |
| North Central | 0.885 | [0.404,1.937] |  | 0.353* | [0.143,0.871] |
| **Type of Place of Residence** |  |  |  |  |  |
| Urban | 1.000 |  |  | 1.000 |  |
| Rural | 1.156 | [0.677,1.975] |  | 0.519 | [0.252,1.070] |
| **No. of living sons** |  |  |  | 1.072 | [0.772,1.490] |
| **No. of FP message channels** |  |  |  | 1.207 | [0.753,1.935] |
|  |  |  |  |  |  |
| **Number of women** | **631** | |  | **631** | |

FP Family planning

* p<0.05; ** p<0.01; *** p<0.001

S2 Appendix. Adjusted odds ratios and 95% confidence intervals for full regression models of the association between child marriage and maternal health outcomes, currently married women age 20-24, Liberia 2019-2020

|  | **Four or More ANC Visits** | |  | **Skilled Attendant at Birth** | |  | **Institutional Delivery** | |
| --- | --- | --- | --- | --- | --- | --- | --- | --- |
| **Characteristics** | **AOR** | **95% CI** |  | **AOR** | **95% CI** |  | **AOR** | **95% CI** |
| **Age at first marriage** |  |  |  |  |  |  |  |  |
| Age 18 and older | 1.000 |  |  | 1.000 |  |  | 1.000 |  |
| Age 15-17 | 1.131 | [0.430,2.976] |  | 0.344*** | [0.184,0.643] |  | 0.582 | [0.259,1.308] |
| Age <15 | 0.520 | [0.151,4.276] |  | 0.361* | [0.142,0.921] |  | 0.702 | [0.284,1.736] |
| **No. of decisions woman made alone or with husband** |  |  |  |  |  |  |  |  |
| **None** | 1.000 |  |  | 1.000 |  |  | 1.000 |  |
| 1 | 0.803 | [0.151,4.277] |  | 1.644 | [0.519,5.206] |  | 0.636 | [0.153,2.648] |
| 2 | 0.942 | [0.245,3.625] |  | 0.495 | [0.168,1.458] |  | 0.427 | [0.108,1.686] |
| 3 | 0.990 | [0.303,3.242] |  | 1.170 | [0.421,3.251] |  | 0.759 | [0.217,2.653] |
| **Woman has right to refuse sex if husband has an STI** |  |  |  |  |  |  |  |  |
| No | 1.000 |  |  | 1.000 |  |  | 1.000 |  |
| Yes | 0.593 | [0.265,1.326] |  | 0.451 | [0.198,1.027] |  | 0.690 | [0.291,1.638] |
| **Spouses’ relative education** |  |  |  |  |  |  |  |  |
| Same/woman higher | 1.000 |  |  | 1.000 |  |  | 1.000 |  |
| Husband higher | 0.766 | [0.351,1.674] |  | 1.034 | [0.588,1.821] |  | 0.984 | [0.495,1.956] |
| **Spouses’ relative age** |  |  |  |  |  |  |  |  |
| < 5 years | 1.000 |  |  | 1.000 |  |  | 1.000 |  |
| Husband 5-9 years older | 0.515 | [0.182,1.462] |  | 0.951 | [0.469,1.930] |  | 0.562 | [0.261,1.208] |
| Husband 10+ years older | 0.574 | [0.191,1.720] |  | 1.390 | [0.629,3.073] |  | 1.113 | [0.428,2.898] |
| **Woman’s age** | 1.053 | [0.758,1.462] |  | 0.651*** | [0.516,0.821] |  | 0.745* | [0.565,0.982] |
| **Woman’s education** |  |  |  |  |  |  |  |  |
| None | 1.000 |  |  | 1.000 |  |  | 1.000 |  |
| Primary | 1.470 | [0.577,3.743] |  | 1.627 | [0.814,3.254] |  | 1.406 | [0.579,3.411] |
| Secondary/higher | 0.738 | [0.698,1.693] |  | 3.102* | [1.214,7.925] |  | 2.626 | [0.914,7.547] |
| **Household wealth** |  |  |  |  |  |  |  |  |
| Low | 1.000 |  |  | 1.000 |  |  | 1.000 |  |
| Medium | 1.241 | [0.782,1.970] |  | 0.741 | [0.387,1.420] |  | 0.727 | [0.309,1.708] |
| High | 1.189 | [0.721, 1.960] |  | 2.250 | [0.909,5.569] |  | 3.469* | [1.018,11.824 |
| **Religion** |  |  |  |  |  |  |  |  |
| Non-Muslim | 1.000 |  |  | 1.000 |  |  | 1.000 |  |
| Muslim | 1.081 | [0.653,1.788] |  | 1.379 | [0.472,4.031] |  | 0.676 | [0.219,2.087] |
| **Region** |  |  |  |  |  |  |  |  |
| Northwestern | 1.000 |  |  | 1.000 |  |  | 1.000 |  |
| South Central | 1.141 | [0.587,2.219] |  | 0.883 | [0.295,2.646] |  | 0.255* | [0.078,0.835] |
| Southeastern A | 0.657 | [0.378,1.142] |  | 1.734 | [0.554,5.427] |  | 0.689 | [0.158,2.999] |
| Southeastern B | 0.422** | [0.236,0.757] |  | 0.915 | [0.298,2.810] |  | 0.663 | [0.202,2.175] |
| North Central | 0.250*** | [0.118,0.531] |  | 2.120 | [0.844,5.328] |  | 2.218 | [0.647,7.608] |
| **Type of Place of Residence** |  |  |  |  |  |  |  |  |
| Urban | 1.000 |  |  | 1.000 |  |  | 1.000 |  |
| Rural | 0.590 | [0.251,1.385] |  | 0.595 | [0.286,1.234] |  | 0.361* | [0.163,0.801] |
| **Birth order** | 0.986 | [0.561,1.736] |  | 2.671*** | [1.806,3.951] |  | 1.441 | [0.941,2.207] |
|  |  |  |  |  |  |  |  |  |
| **Number of women** | **528** | |  | **528** | |  | **528** | |

* p<0.05, ** p<0.01, * ** p<0.001

S3 Appendix. Adjusted odds ratios and 95% confidence intervals for full regression models of the association between child marriage and reproductive health outcomes, currently married women age 20-24, Sierra Leone 2019

|  | **Early Fertility** | |  | **High Fertility** | |  | **Low Fertility Control** | |
| --- | --- | --- | --- | --- | --- | --- | --- | --- |
| **Characteristics** | **AOR** | **95% CI** |  | **AOR** | **95% CI** |  | **AOR** | **95% CI** |
| **Age at first marriage** |  |  |  |  |  |  |  |  |
| Age 18 and older | 1.000 |  |  | 1.000 |  |  | 1.000 |  |
| Age 15-17 | 0.485*** | [0.369,0.638] |  | 3.929*** | [2.397,6.439] |  | 1.728* | [1.139,2.622] |
| Age <15 | 0.347*** | [0.236,0.512] |  | 10.863*** | [6.436,18.336] |  | 3.447*** | [2.131,5.575] |
| **No. of decisions woman made alone or with husband/partner** |  |  |  |  |  |  |  |  |
| None | 1.000 |  |  | 1.000 |  |  | 1.000 |  |
| 1 | 0.918 | [0.628,1.343] |  | 0.313*** | [0.167,0.585] |  | 0.372** | [0.184,0.751] |
| 2 | 1.382 | [0.878,2.175] |  | 0.665 | [0.358,1.235] |  | 0.724 | [0.382,1.373] |
| 3 | 1.106 | [0.825,1.482] |  | 0.686 | [0.446,1.057] |  | 0.689 | [0.453,1.049] |
| **Woman has right to refuse sex if husband has an STI** |  |  |  |  |  |  |  |  |
| No | 1.000 |  |  | 1.000 |  |  | 1.000 |  |
| Yes | 0.988 | [0.729,1.338] |  | 0.867 | [0.599,1.255] |  | 1.077 | [0.727,1.594] |
| **Spouses’ relative education** |  |  |  |  |  |  |  |  |
| Same/woman higher | 1.000 |  |  | 1.000 |  |  | 1.000 |  |
| Husband higher | 1.254 | [0.919,1.710] |  | 1.217 | [0.822,1.801] |  | 1.295 | [0.907,1.849] |
| **Spouses’ relative age** |  |  |  |  |  |  |  |  |
| < 5 years | 1.000 |  |  | 1.000 |  |  | 1.000 |  |
| Husband 5-9 years older | 0.846 | [0.587,1.219] |  | 0.815 | [0.496,1.340] |  | 0.806 | [0.489,1.329] |
| Husband 10+ years older | 0.702* | [0.505,0.975] |  | 1.092 | [0.673,1.772] |  | 0.817 | [0.514,1.299] |
| **Woman’s age** | 0.976 | [0.890,1.071] |  | 1.784*** | [1.544,2.060] |  | 1.319*** | [1.161,1.500] |
| **Woman’s education** |  |  |  |  |  |  |  |  |
| None | 1.000 |  |  | 1.000 |  |  | 1.000 |  |
| Primary | 1.108 | [0.764,1.606] |  | 1.856* | [1.140,3.021] |  | 1.044 | [0.647,1.685] |
| Secondary/higher | 1.186 | [0.853,1.650] |  | 0.712 | [0.461,1.100] |  | 0.643 | [0.401,1.032] |
| **Household wealth** |  |  |  |  |  |  |  |  |
| Low | 1.000 |  |  | 1.000 |  |  | 1.000 |  |
| Medium | 0.975 | [0.717,1.325] |  | 0.705 | [0.444,1.122] |  | 0.858 | [0.573,1.283] |
| High | 0.930 | [0.654,1.322] |  | 0.506** | [0.311,0.824] |  | 0.741 | [0.464,1.184] |
| **Religion** |  |  |  |  |  |  |  |  |
| Non-Muslim | 1.000 |  |  | 1.000 |  |  | 1.000 |  |
| Muslim | 0.739 | [0.514,1.061] |  | 1.389 | [0.876,2.204] |  | 0.908 | [0.581,1.419] |
| **Region** |  |  |  |  |  |  |  |  |
| Eastern | 1.000 |  |  | 1.000 |  |  | 1.000 |  |
| Northern | 1.064 | [0.699,1.619] |  | 0.961 | [0.573,1.611] |  | 0.912 | [0.530,1.569] |
| Northwestern | 1.484 | [0.941,2.341] |  | 1.204 | [0.693,2.093] |  | 0.930 | [0.505,1.716] |
| Southern | 1.525* | [1.027,2.266] |  | 1.181 | [0.707,1.973] |  | 1.201 | [0.745,1.936] |
| Western | 1.189 | [0.693,2.038] |  | 1.251 | [0.557,2.808] |  | 0.735 | [0.333,1.624] |
| **Type of Place of Residence** |  |  |  |  |  |  |  |  |
| Urban | 1.000 |  |  | 1.000 |  |  | 1.000 |  |
| Rural | 1.279 | [0.818,1.998] |  | 2.717*** | [1.625,4.542] |  | 1.537 | [0.818,2.887] |
|  |  |  |  |  |  |  |  |  |
| **Number of women** | **1,325** | |  | **1,325** | |  | **1,325** | |

* p<0.05, ** p<0.01, * ** p<0.001

S3 Appendix Contd.

|  | **Unwanted/Mistimed Pregnancy** | |  | **Modern Contraceptive Use** | |
| --- | --- | --- | --- | --- | --- |
| **Characteristics** | **AOR** | **95% CI** |  | **AOR** | **95% CI** |
| **Age at first marriage** |  |  |  |  |  |
| Age 18 and older | 1.000 |  |  | 1.000 |  |
| Age 15-17 | 1.088 | [0.720,1.644] |  | 1.314 | [0.847,2.038] |
| Age <15 | 0.786 | [0.440,1.405] |  | 2.138** | [1.252,3.650] |
| **No. of decisions woman made alone or with husband/partner** |  |  |  |  |  |
| None | 1.000 |  |  | 1.000 |  |
| 1 | 1.634 | [0.950,2.810] |  | 1.461 | [0.823,2.594] |
| 2 | 1.906* | [1.017,3.572] |  | 1.522 | [0.851,2.723] |
| 3 | 0.651 | [0.398,1.064] |  | 1.332 | [0.898,1.976] |
| **Woman has right to refuse sex if husband has an STI** |  |  |  |  |  |
| No | 1.000 |  |  | 1.000 |  |
| Yes | 0.861 | [0.567,1.308] |  | 2.338*** | [1.473,3.712] |
| **Spouses’ relative education** |  |  |  |  |  |
| Same/woman higher | 1.000 |  |  | 1.000 |  |
| Husband higher | 1.266 | [0.836,1.918] |  | 1.423 | [0.992,2.042] |
| **Spouses’ relative age** |  |  |  |  |  |
| < 5 years | 1.000 |  |  | 1.000 |  |
| Husband 5-9 years older | 0.759 | [0.464,1.242] |  | 0.999 | [0.632,1.578] |
| Husband 10+ years older | 0.670 | [0.403,1.114] |  | 0.694 | [0.444,1.084] |
| **Woman’s age** | 1.032 | [0.893,1.192] |  | 1.109 | [0.989,1.244] |
| **Woman’s education** |  |  |  |  |  |
| None | 1.000 |  |  | 1.000 |  |
| Primary | 1.264 | [0.716,2.231] |  | 1.356 | [0.763,2.410] |
| Secondary/higher | 2.298** | [1.340,3.941] |  | 2.530*** | [1.599,4.003] |
| **Household wealth** |  |  |  |  |  |
| Low | 1.000 |  |  | 1.000 |  |
| Medium | 0.778 | [0.480,1.260] |  | 1.395 | [0.892,2.181] |
| High | 0.731 | [0.432,1.237] |  | 1.872* | [1.153,3.041] |
| **Religion** |  |  |  |  |  |
| Non-Muslim | 1.000 |  |  | 1.000 |  |
| Muslim | 0.902 | [0.588,1.382] |  | 0.731 | [0.462,1.157] |
| **Region** |  |  |  |  |  |
| Eastern | 1.000 |  |  | 1.000 |  |
| Northern | 1.067 | [0.566,2.011] |  | 0.899 | [0.533,1.517] |
| Northwestern | 1.570 | [0.828,2.977] |  | 0.625 | [0.354,1.103] |
| Southern | 1.361 | [0.748,2.477] |  | 1.299 | [0.789,2.138] |
| Western | 4.128*** | [2.103,8.105] |  | 0.663 | [0.337,1.304] |
| **Type of Place of Residence** |  |  |  |  |  |
| Urban | 1.000 |  |  | 1.000 |  |
| Rural | 2.339** | [1.352,4.046] |  | 0.535* | [0.327,0.877] |
| No. of living sons |  |  |  | 0.239 | [0.984,1.561] |
| No. of FP message channels |  |  |  | 0.069 | [0.790,1.448] |
|  |  |  |  |  |  |
| **Number of women** | **1,325** | |  | **1,325** | |

FP Family planning

* p<0.05, ** p<0.01, * ** p<0.001

S4 Appendix. Adjusted odds ratios and 95% confidence intervals for full regression models of the association between child marriage and maternal health outcomes, currently married women age 20-24, Sierra Leone 2019

|  | **Four or More ANC Visits** | |  | **Skilled Attendant at Birth** | |  | **Institutional Delivery** | |
| --- | --- | --- | --- | --- | --- | --- | --- | --- |
| **Characteristics** | **AOR** | **95% CI** |  | **AOR** | **95% CI** |  | **AOR** | **95% CI** |
| **Age at first marriage** |  |  |  |  |  |  |  |  |
| Age 18 and older | 1.000 |  |  | 1.000 |  |  | 1.000 |  |
| Age 15-17 | 1.090 | [0.756,1.572] |  | 1.170 | [0.670,2.040] |  | 1.051 | [0.653,1.693] |
| Age <15 | 0.926 | [0.541,1.585] |  | 1.213 | [0.612,2.401] |  | 1.043 | [0.567,1.919] |
| **No. of decisions woman made alone or with husband/partner** |  |  |  |  |  |  |  |  |
| None | 1.000 |  |  | 1.000 |  |  | 1.000 |  |
| 1 | 0.761 | [0.453,1.278] |  | 0.773 | [0.411,1.452] |  | 0.573* | [0.330,0.996] |
| 2 | 0.818 | [0.467,1.431] |  | 1.774 | [0.725,4.343] |  | 1.188 | [0.556,2.536] |
| 3 | 1.015 | [0.634,1.624] |  | 1.170 | [0.682, 2.007] |  | 1.053 | [0.645,1.719] |
| **Woman has right to refuse sex if husband has an STI** |  |  |  |  |  |  |  |  |
| No | 1.000 |  |  | 1.000 |  |  | 1.000 |  |
| Yes | 1.393 | [0.956,2.031] |  | 0.607* | [0.383, .960] |  | 0.538** | [0.353,0.820] |
| **Spouses’ relative education** |  |  |  |  |  |  |  |  |
| Same/woman higher | 1.000 |  |  | 1.000 |  |  | 1.000 |  |
| Husband higher | 0.938 | [0.632,1.393] |  | 1.318 | [0.722,2.407] |  | 1.087 | [0.651,1.815] |
| **Spouses’ relative age** |  |  |  |  |  |  |  |  |
| < 5 years | 1.000 |  |  | 1.000 |  |  | 1.000 |  |
| Husband 5-9 years older | 0.763 | [0.447,1.303 |  | 1.071 | [0.583,1.968] |  | 0.993 | [0.581,1.695] |
| Husband 10+ years older | 0.716 | [0.453,1.130] |  | 1.106 | [0.604,2.022] |  | 1.051 | [0.604,1.829] |
| **Woman’s age** | 0.945 | [0.828,1.077] |  | 0.967 | [0.799,1.171] |  | 0.934 | [0.797,1.093] |
| **Woman’s education** |  |  |  |  |  |  |  |  |
| None | 1.000 |  |  | 1.000 |  |  | 1.000 |  |
| Primary | 0.785 | [0.476,1.295] |  | 1.469 | 0.871, 2.478] |  | 1.676 | [0.992,2.830] |
| Secondary/higher | 1.087 | [0.698,1.693] |  | 2.906*** | [1.591, 5.308] |  | 2.817*** | [1.668,4.757] |
| **Household wealth** |  |  |  |  |  |  |  |  |
| Low | 1.000 |  |  | 1.000 |  |  | 1.000 |  |
| Medium | 1.241 | [0.782,1.970] |  | 0.916 | [0.535,1.568] |  | 0.948 | [0.584,1.540] |
| High | 1.189 | [0.721, 1.960] |  | 0.867 | [0.444,1.693] |  | 1.265 | [0.673,2.378] |
| **Religion** |  |  |  |  |  |  |  |  |
| Non-Muslim | 1.000 |  |  | 1.000 |  |  | 1.000 |  |
| Muslim | 1.081 | [0.653,1.788] |  | 1.063 | [0.548, 2.061] |  | 1.04 | [0.542,1.995] |
| **Region** |  |  |  |  |  |  |  |  |
| Eastern | 1.000 |  |  | 1.000 |  |  | 1.000 |  |
| Northern | 1.141 | [0.587,2.219] |  | 0.396* | [0.175,0.895] |  | 0.295*** | [0.144,0.606] |
| Northwestern | 0.657 | [0.378,1.142] |  | 0.195*** | [0.086,0.439] |  | 0.207*** | [0.099,0.433] |
| Southern | 0.422** | [0.236,0.757] |  | 0.636 | [0.256,1.584] |  | 0.798 | [0.324,1.969] |
| Western | 0.250*** | [0.118,0.531] |  | 0.360 | [0.118,1.100] |  | 0.269** | [0.110,0.661] |
| **Type of Place of Residence** |  |  |  |  |  |  |  |  |
| Urban | 1.000 |  |  | 1.000 |  |  | 1.000 |  |
| Rural | 1.042 | [0.846,1.347] |  | 0.373* | [0.161,0.862] |  | 0.755 | [0.418,1.363] |
| Birth order | 1.068 | [0.860,1.347] |  | 0.872 | [0.626,1.213] |  | 0.917 | [0.691,1.218] |
|  |  |  |  |  |  |  |  |  |
| **Number of women** | **1,093** | |  | **1,093** | |  | **1,093** | |

FP Family planning

* p<0.05, ** p<0.01, * ** p<0.001

**COUNTRY DIFFERENCES IN OUTCOMES**

Table 7 Adjusted odds ratios and 95% confidence intervals for the association between child marriage and reproductive health outcomes after adjusting for other factors, currently married women age 20-24, Sierra Leone 2019 and Liberia 2019-2020 Combined

|  | **Early Fertility** | |  | **High Fertility** | |  | **Low Fertility Control** | |  | **Has Had a Terminated Pregnancy** | |
| --- | --- | --- | --- | --- | --- | --- | --- | --- | --- | --- | --- |
| **Characteristics** | **AOR** | **95% CI** |  | **AOR** | **95% CI** |  | **AOR** | **95% CI** |  |  |  |
| **Age at first marriage** |  |  |  |  |  |  |  |  |  |  |  |
| Age 18 and older | 1.000 |  |  | 1.000 |  |  | 1.000 |  |  | 1.000 |  |
| Age 15-17 | 0.543*** | [0.428,0.691] |  | 3.620*** | [2.405,5.448] |  | 1.696** | [1.202,2.393] |  | 0.804 | [0.521,1.240] |
| Age <15 | 0.325*** | [0.233,0.455] |  | 10.673*** | [7.128,15.980] |  | 3.198*** | [2.178,4.695] |  | 0.918 | [0.576,1.464] |
| **Country** |  |  |  |  |  |  |  |  |  |  |  |
| Liberia | 1.000 |  |  | 1.000 |  |  | 1.000 |  |  | 1.000 |  |
| Sierra Leone | 0.914 | [0.660,1.266] |  | 0.736 | [0.478,1.133] |  | 0.696 | [0.429,1.129] |  | 0.576* | [0.349,0.951] |
| **No. of decisions woman made alone or with husband/partner** |  |  |  |  |  |  |  |  |  |  |  |
| None | 1.000 |  |  | 1.000 |  |  | 1.000 |  |  | 1.000 |  |
| 1 | 0.875 | [0.631,1.214] |  | 0.324*** | [0.187,0.561] |  | 0.414** | [0.235,0.728] |  | 1.277 | [0.716,2.277] |
| 2 | 1.364 | [0.937,1.984] |  | 0.597* | [0.357,1.000] |  | 0.517* | [0.296,0.902] |  | 1.273 | [0.693,2.339] |
| 3 | 1.198 | [0.916,1.568] |  | 0.769 | [0.537,1.100] |  | 0.664* | [0.462,0.954] |  | 1.336 | [0.837,2.132] |
| **Woman has right to refuse sex if husband has an STI** |  |  |  |  |  |  |  |  |  |  |  |
| No | 1.000 |  |  | 1.000 |  |  | 1.000 |  |  | 1.000 |  |
| Yes | 1.024 | [0.782,1.341] |  | 0.846 | [0.592,1.208] |  | 1.219 | [0.880,1.690] |  | 1.350 | [0.769,2.369] |
| **Spouses’ relative education** |  |  |  |  |  |  |  |  |  |  |  |
| Same/woman higher | 1.000 |  |  | 1.000 |  |  | 1.000 |  |  | 1.000 |  |
| Husband higher | 1.09 | [0.844,1.407] |  | 0.964 | [0.673,1.381] |  | 1.065 | [0.754,1.506] |  | 1.168 | [0.766,1.780] |
| **Spouses’ relative age** |  |  |  |  |  |  |  |  |  |  |  |
| < 5 years | 1.000 |  |  | 1.000 |  |  | 1.000 |  |  | 1.000 |  |
| Husband 5-9 years older | 0.936 | [0.677,1.295] |  | 1.031 | [0.672,1.581] |  | 1.067 | [0.709,1.607] |  | 0.967 | [0.600,1.559] |
| Husband 10+ years older | 0.818 | [0.615,1.086] |  | 1.375 | [0.941,2.011] |  | 1.140 | [0.746,1.742] |  | 1.145 | [0.707,1.854] |
| **Woman’s age** | 1.004 | [0.931,1.083] |  | 1.865*** | [1.649,2.108] |  | 1.288*** | [1.155,1.436] |  | 1.065 | [0.924,1.227] |
| **Woman’s education** |  |  |  |  |  |  |  |  |  |  |  |
| None | 1.000 |  |  | 1.000 |  |  | 1.000 |  |  | 1.000 |  |
| Primary | 1.057 | [0.774,1.442] |  | 1.695** | [1.145,2.508] |  | 1.104 | [0.738,1.651] |  | 1.108 | [0.634,1.936] |
| Secondary/higher | 1.043 | [0.775,1.404] |  | 0.704 | [0.486,1.021] |  | 0.754 | [0.505,1.128] |  | 0.596 | [0.346,1.028] |
| **Household wealth** |  |  |  |  |  |  |  |  |  |  |  |
| Low | 1.000 |  |  | 1.000 |  |  | 1.000 |  |  | 1.000 |  |
| Medium | 0.893 | [0.688,1.159] |  | 0.713 | [0.489,1.041] |  | 0.752 | [0.528,1.071] |  | 0.827 | [0.531,1.286] |
| High | 0.894 | [0.669,1.195] |  | 0.434*** | [0.286,0.659] |  | 0.614* | [0.420,0.900] |  | 0.931 | [0.595,1.457] |
| **Religion** |  |  |  |  |  |  |  |  |  |  |  |
| Non-Muslim | 1.000 |  |  | 1.000 |  |  | 1.000 |  |  | 1.000 |  |
| Muslim | 0.827 | [0.613,1.115] |  | 1.296 | [0.880,1.910] |  | 1.032 | [0.691,1.542] |  | 1.078 | [0.678,1.715] |
| **Type of place of residence** |  |  |  |  |  |  |  |  |  |  |  |
| Urban | 1.000 |  |  | 1.000 |  |  | 1.000 |  |  | 1.000 |  |
| Rural | 1.330* | [1.007,1.758] |  | 2.737*** | [1.938,3.865] |  | 2.020*** | [1.350,3.021] |  | 0.593* | [0.383,0.919] |
|  |  |  |  |  |  |  |  |  |  |  |  |
| **Number of Cases** | **1997** |  |  | **1997** |  |  | **1997** |  |  | **1997** |  |

Note: All regression models control for region.

* p<0.05, ** p<0.01, * ** p<0.001

Table 8 Adjusted odds ratios and 95% confidence intervals for the association between child marriage and reproductive health outcomes after adjusting for other factors, currently married women age 20-24, Sierra Leone 2019 and Liberia 2019-2020 Combined

|  | **Unwanted Pregnancy** | |  | **Multiple Unwanted Pregnancy** | |  | **Modern Contraceptive Use** | |
| --- | --- | --- | --- | --- | --- | --- | --- | --- |
| **Characteristics** | **AOR** | **95% CI** |  | **AOR** | **95% CI** |  | **AOR** | **95% CI** |
| **Age at first marriage** |  |  |  |  |  |  |  |  |
| Age 18 and older | 1.000 |  |  | 1.000 |  |  | 1.000 |  |
| Age 15-17 | 1.104 | [0.767,1.589] |  | 2.597 | [0.882,7.642] |  | 1.336 | [0.959,1.862] |
| Age <15 | 0.732 | [0.472,1.134] |  | 1.219 | [0.365,4.065] |  | 2.050*** | [1.367,3.074] |
| **Country** |  |  |  |  |  |  |  |  |
| Liberia | 1.000 |  |  | 1.000 |  |  | 1.000 |  |
| Sierra Leone | 0.356*** | [0.242,0.525] |  | 0.139*** | [0.058,0.333] |  | 0.816 | [0.538,1.239] |
| **No. of decisions woman made alone or with husband** |  |  |  |  |  |  |  |  |
| **None** | 1.000 |  |  | 1.000 |  |  | 1.000 |  |
| 1 | 1.565 | [0.974,2.515] |  | 4.918* | [1.234,19.606] |  | 1.274 | [0.784,2.070] |
| 2 | 1.563 | [0.932,2.621] |  | 1.952 | [0.697,5.468] |  | 1.343 | [0.830,2.175] |
| 3 | 0.899 | [0.622,1.299] |  | 1.159 | [0.435,3.089] |  | 1.342 | [0.946,1.903] |
| **Woman has right to refuse sex if husband has an STI** |  |  |  |  |  |  |  |  |
| No | 1.000 |  |  | 1.000 |  |  | 1.000 |  |
| Yes | 0.922 | [0.661,1.287] |  | 0.801 | [0.303,2.114] |  | 1.852*** | [1.297,2.645] |
| **Spouses’ relative education** |  |  |  |  |  |  |  |  |
| Same/woman higher | 1.000 |  |  | 1.000 |  |  | 1.000 |  |
| Husband higher | 1.028 | [0.742,1.424] |  | 0.707 | [0.347,1.440] |  | 1.279 | [0.956,1.711] |
| **Spouses’ relative age** |  |  |  |  |  |  |  |  |
| < 5 years | 1.000 |  |  | 1.000 |  |  | 1.000 |  |
| Husband 5-9 years older | 0.887 | [0.631,1.245] |  | 2.100* | [1.121,3.935] |  | 0.988 | [0.697,1.401] |
| Husband 10+ years older | 0.618** | [0.430,0.887] |  | 0.914 | [0.379,2.205] |  | 0.674* | [0.461,0.985] |
| **Woman’s age** | 0.991 | [0.899,1.092] |  | 1.218 | [0.972,1.526] |  | 1.025 | [0.934,1.126] |
| **Woman’s education** |  |  |  |  |  |  |  |  |
| None | 1.000 |  |  | 1.000 |  |  | 1.000 |  |
| Primary | 1.566* | [1.025,2.394] |  | 3.482* | [1.076,11.271] |  | 1.357 | [0.902,2.044 |
| Secondary/higher | 2.241*** | [1.470,3.417] |  | 3.931* | [1.238,12.479] |  | 1.955*** | [1.326,2.881 |
| **Household wealth** |  |  |  |  |  |  |  |  |
| Low | 1.000 |  |  | 1.000 |  |  | 1.000 |  |
| Medium | 1.055 | [0.747,1.491] |  | 0.952 | [0.502,1.805] |  | 1.709** | [1.205,2.423] |
| High | 0.959 | [0.656,1.402] |  | 0.557 | [0.237,1.309] |  | 1.620* | [1.103,2.380] |
| **Religion** |  |  |  |  |  |  |  |  |
| Non-Muslim | 1.000 |  |  | 1.000 |  |  | 1.000 |  |
| Muslim | 0.883 | [0.608,1.284] |  | 0.653 | [0.295,1.442] |  | 0.614* | [0.421,0.895] |
| **Type of Place of Residence** |  |  |  |  |  |  |  |  |
| Urban | 1.000 |  |  | 1.000 |  |  | 1.000 |  |
| Rural | 1.105 | [0.808,1.510] |  | 1.166 | [0.497,2.733] |  | 0.529*** | [0.367,0.762] |
| **No. of living sons** |  |  |  |  |  |  | 1.183 | [0.987,1.418] |
| **No. of FP message channels** |  |  |  |  |  |  | 1.167 | [0.926,1.470] |
|  |  |  |  |  |  |  |  |  |
| **Number of Cases** | **1997** |  |  | **1997** |  |  | **1997** |  |

Note: All regression models control for region.

* p<0.05, ** p<0.01, * ** p<0.001

Table 9 Adjusted odds ratios and 95% confidence intervals for the association between child marriage and maternal health outcomes after adjusting for other factors, currently married women age 20-24, Sierra Leone 2019 and Liberia 2019-2020 Combined

|  | **Four or More ANC Visits** | |  | **Skilled Attendant at Birth** | |  | **Institutional Delivery** | |
| --- | --- | --- | --- | --- | --- | --- | --- | --- |
| **Characteristics** | **AOR** | **95% CI** |  | **AOR** | **95% CI** |  | **AOR** | **95% CI** |
| **Age at first marriage** |  |  |  |  |  |  |  |  |
| Age 18 and older | 1.000 |  |  | 1.000 |  |  | 1.000 |  |
| Age 15-17 | 0.838 | [0.537,1.310] |  | 0.575** | [0.403,0.820] |  | 0.866 | [0.578,1.299] |
| Age <15 | 0.553 | [0.304,1.008] |  | 0.361*** | [0.222,0.587] |  | 0.944 | [0.574,1.552] |
| **Country** |  |  |  |  |  |  |  |  |
| Liberia | 1.000 |  |  | 1.000 |  |  | 1.000 |  |
| Sierra Leone | 0.976 | [0.521,1.827] |  | 1.022 | [0.674,1.550] |  | 1.113 | [0.649,1.908] |
| **No. of decisions woman made alone or with husband/partner** |  |  |  |  |  |  |  |  |
| None | 1.000 |  |  | 1.000 |  |  | 1.000 |  |
| 1 | 1.038 | [0.560,1.926] |  | 1.011 | [0.652,1.569] |  | 0.622 | [0.374,1.033] |
| 2 | 1.563 | [0.852,2.868] |  | 1.093 | [0.642,1.862] |  | 0.911 | [0.492,1.687] |
| 3 | 1.671 | [0.986,2.832] |  | 1.107 | [0.770,1.589] |  | 1.021 | [0.676,1.542] |
| **Woman has right to refuse sex if husband has an STI** |  |  |  |  |  |  |  |  |
| No | 1.000 |  |  | 1.000 |  |  | 1.000 |  |
| Yes | 1.041 | [0.676,1.603] |  | 0.480*** | [0.334,0.689] |  | 0.544** | [0.376,0.786] |
| **Spouses’ relative education** |  |  |  |  |  |  |  |  |
| Same/woman higher | 1.000 |  |  | 1.000 |  |  | 1.000 |  |
| Husband higher | 1.058 | [0.662,1.692] |  | 1.023 | [0.737,1.421] |  | 1.020 | [0.688,1.514] |
| **Spouses’ relative age** |  |  |  |  |  |  |  |  |
| < 5 years | 1.000 |  |  | 1.000 |  |  | 1.000 |  |
| Husband 5-9 years older | 0.779 | [0.449,1.353] |  | 1.214 | [0.797,1.848] |  | 0.827 | [0.531,1.288] |
| Husband 10+ years older | 0.779 | [0.471,1.288] |  | 1.248 | [0.810,1.922] |  | 1.097 | [0.688,1.750] |
| **Woman’s age** | 1.034 | [0.875,1.222] |  | 0.767*** | [0.676,0.869] |  | 0.851* | [0.744,0.974] |
| **Woman’s education** |  |  |  |  |  |  |  |  |
| None | 1.000 |  |  | 1.000 |  |  | 1.000 |  |
| Primary | 0.797 | [0.464,1.369] |  | 1.145 | [0.801,1.635] |  | 1.597* | [1.074,2.374] |
| Secondary/higher | 0.955 | [0.586,1.556] |  | 2.513*** | [1.696,3.725] |  | 2.827*** | [1.809,4.418] |
| **Household wealth** |  |  |  |  |  |  |  |  |
| Low | 1.000 |  |  | 1.000 |  |  | 1.000 |  |
| Medium | 1.279 | [0.763,2.141] |  | 0.758 | [0.530,1.084] |  | 0.795 | [0.531,1.191] |
| High | 1.418 | [0.784,2.566] |  | 0.937 | [0.636,1.381] |  | 1.294 | [0.804,2.083] |
| **Religion** |  |  |  |  |  |  |  |  |
| Non-Muslim | 1.000 |  |  | 1.000 |  |  | 1.000 |  |
| Muslim | 1.056 | [0.613,1.818] |  | 1.280 | [0.863,1.898] |  | 1.007 | [0.588,1.727] |
| **Type of place of residence** |  |  |  |  |  |  |  |  |
| Urban | 1.000 |  |  | 1.000 |  |  | 1.000 |  |
| Rural | 0.981 | [0.594,1.621] |  | 0.757 | [0.514,1.117] |  | 0.803 | [0.524,1.230] |
| **Birth order** | 0.972 | [0.770,1.228] |  | 1.889*** | [1.438,2.482] |  | 1.053 | [0.839,1.323] |
|  |  |  |  |  |  |  |  |  |
| **Number of Cases** | **1521** |  |  | **1789** |  |  | **1655** |  |

Note: All regression models control for region.

FP Family planning

* p<0.05, ** p<0.01, * ** p<0.001

Adjusted odds ratios and confidence intervals from regressions of the association of child marriage with reproductive and maternal health outcomes, currently married women age 20-24, Sierra Leone 2019 and Liberia 2019-2020

|  | **Liberia** | |  | **Sierra Leone** | |
| --- | --- | --- | --- | --- | --- |
| **Outcome** | **15-17** | **<15** |  | **15-17** | **<15** |
| Early fertility | 0.61 [0.36,1.03] | 0.27** [0.12,0.59] |  | 0.49*** 0.37,0.64] | 0.347*** [0.24,0.5] |
| High fertility | 2.73** [1.29,5.82] | 13.13*** [5.86,29.45] |  | 3.93*** [2.40,6.44] | 10.86***[6.44,18.34] |
| Low fertility control | 1.69 [0.87,3.28] | 3.94*** [1.91,8.12] |  | 1.73* [1.14,2.62] | 3.45*** [2.13,5.58] |
| Ever had terminated pregnancy | 0.92 [0.43,1.97] | 0.74 [0.31,1.79] |  | 0.67 [0.39,1.14] | 0.98 [0.54,1.78] |
| Unwanted pregnancy | 1.21 [0.60,2.42] | 0.85 [0.37,1.97] |  | 1.09 [0.72,1.64] | 0.79 [0.44,1.41] |
| Multiple unwanted pregnancies | 2.258 [0.58,8.84] | 0.78 [0.14,4.42] |  | 3.80 [0.74,19.4] | 3.86 [0.78,19.15] |
| Modern contraceptive use | 1.30 [0.73,2.32] | 1.65 [0.75,3.59] |  | 1.31 [0.85,2.04] | 2.14** [1.25,3.65] |
| Four or more ANC visits ^a^ | 0.72 [0.21,2.47] | 0.31 [0.08,1.19] |  | 0.89 [0.545,1.466] | 0.697 [0.344,1.412] |
| Skilled personnel at delivery ^a^ | 0.34*** [0.18,0.64] | 0.36* [0.14,0.92] |  | 0.74 [0.48,1.14] | 0.37*** [0.21,0.66] |
| Institutional delivery ^a^ | 0.58 [0.26,1.31] | 0.70 [0.28,1.74] |  | 1.05 [0.65,1.69] | 1.04 [0.57,1.92] |

Notes: Data were weighted. All models controlled for age, type of place of residence, household wealth, woman’s level of education, spousal age gap, spousal education gap, religion, ability to negotiate safe sex, and region of residence. For modern contraceptive use, regressions also control for number of living sons, and number of family planning message channels. The age at marriage reference categories is 18 years or older.

^a^ Restricted to women with a live birth in the past five years

* Significant at p < .05; ** p < .005
